# Allosteric modulation of cardiac myosin dynamics by omecamtiv mecarbil

**DOI:** 10.1371/journal.pcbi.1005826

**Published:** 2017-11-06

**Authors:** Shaima Hashem, Matteo Tiberti, Arianna Fornili

**Affiliations:** 1 School of Biological and Chemical Sciences, Queen Mary University of London, London, United Kingdom; 2 The Thomas Young Centre for Theory and Simulation of Materials, London, United Kingdom; Fudan University, CHINA

## Abstract

New promising avenues for the pharmacological treatment of skeletal and heart muscle diseases rely on direct sarcomeric modulators, which are molecules that can directly bind to sarcomeric proteins and either inhibit or enhance their activity. A recent breakthrough has been the discovery of the myosin activator omecamtiv mecarbil (OM), which has been shown to increase the power output of the cardiac muscle and is currently in clinical trials for the treatment of heart failure. While the overall effect of OM on the mechano-chemical cycle of myosin is to increase the fraction of myosin molecules in the sarcomere that are strongly bound to actin, the molecular basis of its action is still not completely clear. We present here a Molecular Dynamics study of the motor domain of human cardiac myosin bound to OM, where the effects of the drug on the dynamical properties of the protein are investigated for the first time with atomistic resolution. We found that OM has a double effect on myosin dynamics, inducing a) an increased coupling of the motions of the converter and lever arm subdomains to the rest of the protein and b) a rewiring of the network of dynamic correlations, which produces preferential communication pathways between the OM binding site and distant functional regions. The location of the residues responsible for these effects suggests possible strategies for the future development of improved drugs and the targeting of specific cardiomyopathy-related mutations.

## Author summary

Cardiac myosin is a motor protein responsible for the contraction of the heart muscle. New strategies for the cure of heart diseases are currently being developed by using myosin modulators, which are small molecules that can interact with myosin and modify its activity. The advantage of this approach over traditional drugs is that by directly targeting cardiac myosin it is possible to have drugs with reduced side effects. Moreover, the availability of a spectrum of molecules to finely tune myosin to a desired level of activity opens the possibility to develop more precise and personalised drug therapies. In this work, we study a recently discovered activator of cardiac myosin, omecamtiv mecarbil, in order to understand its mechanism of action. In particular, we use Molecular Dynamics simulations to unravel the effects of the drug on myosin motions, which are closely related to its function. We find that omecamtiv has a strong effect on myosin dynamics and it changes the way regions of the protein that are critical for its function interact with each other. We use these data to identify genetic mutations associated with heart diseases that could be targeted by the drug and to suggest a possible route to design drugs with different therapeutic properties.

## Introduction

Sarcomeric modulators are small molecules that can modify the function of proteins in the sarcomere, the fundamental repeating unit of skeletal and cardiac muscle cells. New promising avenues for the pharmacological treatment of different muscle and heart diseases rely on direct sarcomeric modulators, which are molecules that can directly bind to sarcomeric proteins and either inhibit or enhance their activity [1]. Part of the current research is focusing on modulators of myosin II, the motor protein responsible for muscle contraction, with different drugs either in preclinical development or in clinical trials [1–3]. The possibility to modulate myosin function by either up or down-regulating it is particularly appealing for the treatment of inherited cardiac diseases. Indeed, myosin mutations are associated with cardiomyopathies with different phenotypes, including hypertrophic (HCM) and dilated cardiomyopathy (DCM), and myosin modulators could be potentially used to counteract their damaging effect, with specific drugs tailored for specific mutations [1, 4, 5].

**Fig 1:**
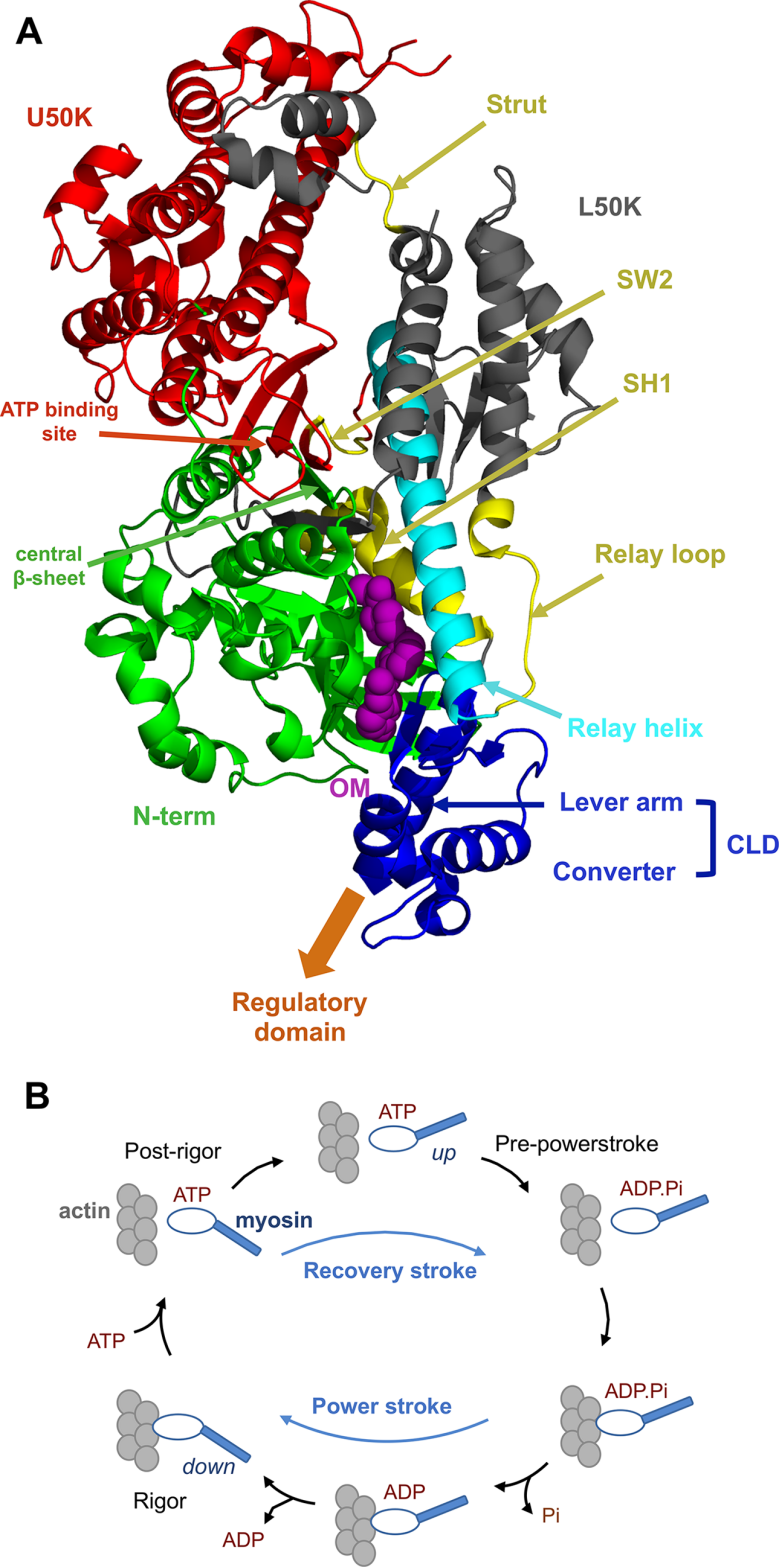
Structure of the myosin motor domain and acto-myosin cycle. A. Cartoon representation of the motor domain structure (PDB ID: 4PA0 [8]), with the subdomains highlighted in different colours and OM shown as purple spheres. The motor domain is connected to the rest of myosin through the lever arm and the regulatory domain (not shown). B. Simplified representation of the acto-myosin cycle, where myosin switches between actin-bound (bottom) and -unbound (top) states and between up and down conformations of the lever arm.

The action of myosin modulators is closely related to the allosteric nature of the protein and in particular of its motor domain (Fig 1A), which is the domain responsible for the hydrolysis of ATP and the conversion of the resulting chemical energy into mechanical work. The motor domain is composed of four main subdomains, namely the N-terminal (N-term, green), the upper 50-K (U50K, red), the lower 50-K (L50K, grey) and the converter (blue) subdomains, connected by linkers (cyan and yellow). The motor domain is then connected to the rest of the myosin molecule through the lever arm helix (blue), which is strongly coupled to the converter. According to most of the current models of the molecular mechanisms at the basis of myosin function, the relative orientation of the subdomains is controlled by the conformation of the linkers, which is in turn regulated by the biochemical state of myosin. In particular, Switch 2 (SW2), the relay helix and the SH1 helix adopt different conformations in the different stages of the acto-myosin cycle, where myosin switches from actin-bound to actin-unbound states according to the nature of the nucleotide bound to it (Fig 1B). The conformational changes occurring in the linkers are propagated and amplified by the reorientation of the subdomains connected to them, so that small changes in the ATP-binding site in the U50K subdomain can ultimately lead to the powerstroke, a large swinging motion of the lever arm that is caused by the rotation of the converter subdomain and is responsible for the production of mechanical work when myosin is bound to actin. The powerstroke conformational changes are reversed upon ATP binding and actin unbinding in the recovery stroke, which is the transition from the post-rigor to the pre-powerstroke state that restores the up position of the lever arm [6, 7].

In addition to actin and ATP binding sites, the motor domain of myosins has different pockets that can be bound by small molecules [3, 9]. Allosteric modulators binding to these sites have been shown to affect myosin function in different ways and using different mechanisms. In particular, myosin II can be targeted by both inhibitors and activators. Most of the myosin II inhibitors discovered so far have been found to decrease the release of inorganic phosphate (Pi) after ATP hydrolysis, the rate limiting step of the acto-myosin cycle [3]. While many of these molecules have been used only as research tools because of their toxicity, the recently developed MYK-461 [5] and MYK-491 have passed the preclinical development stage and are currently in clinical trials [10]. In particular, MYK-461 has been tested in mouse models of HCM, where it is has been shown to slow down the progression of the disease [5]. Structural information on the binding site is available only for some of these inhibitors. Blebbistatin [11] binds to the 50-K cleft between the U50K and L50K subdomains, while the smooth muscle myosin inhibitor CK-571 has been recently shown to bind to a pocket between the relay and SH1 helices [12]. Interestingly, CK-571 uses a unique inhibition mechanism where the drug stabilises a previously unknown intermediate step along the recovery stroke and prevents myosin to reach the pre-powerstroke state, thus hindering the ATP hydrolysis [12].

Myosin activators, namely compounds that enhance myosin activity instead of inhibiting it, have been relatively less studied [3]. A recent breakthrough in the pharmacological treatment of cardiac disease has been the discovery of the myosin activator omecamtiv mecarbil (OM) [13]. The overall effect of OM on the acto-myosin cycle is to increase the duty ratio, which is the fraction of myosin molecules in the sarcomere that are strongly bound to actin. This effect is considered to result from an increase in the rate of transition from weakly to strongly bound states that leads to the faster release of Pi measured in the presence of actin[13]. The larger duty ratio causes an increase in the force produced by the sarcomere (ensemble force) [4], so that the overall effect of OM binding is an increased heart contractility [13]. At the same time, OM has been shown to have an inhibitory effect on the velocity of actin filaments in *in vitro* motility assays [14, 15] and on the powerstroke rate in time resolved FRET experiments [16], while contrasting results have been found when studying its effect on the actin-activated ATP hydrolysis rate [13, 15].

The increased force generation induced by OM makes it suitable for the treatment of heart conditions that are characterised by a decreased cardiac contractility. Indeed, OM is currently due to start phase III clinical trials for the treatment of heart failure [17, 18], after previous investigations showed that it was well tolerated and it had beneficial pharmacological effects such as an increased systolic function, a reduced left ventricular wall stress and a beneficial left ventricular remodelling [19–24]. The trials did not show any evidence of the adverse effects observed with the administration of the drugs currently used to treat heart failure, such as increased heart rate, arrhythmias or hypotension, while the presence of other dose-dependent side effects (increased troponin levels and reduced diastolic filling times) will be further investigated in larger patient cohorts [18].

Understanding the molecular basis of OM action is essential to design strategies for the development of new modulators and their tailoring to specific diseases. The opposing effects of OM on the kinetics of the acto-myosin cycle suggest that its binding has a complex effect on myosin at the atomistic level. Recent structural studies [8] showed that OM binds to a deeply buried pocket between the L50K and N-terminal subdomains, close to the SH1 helix and the converter (Fig 1A). Since these regions are critical for the allosteric communication between the lever arm and the nucleotide binding site, it was suggested that OM can affect the coupling between the lever arm motion and the nucleotide state [8]. Moreover, small differences were found between Apo and OM-bound structures of the central β-sheet. Modifications in this part of the protein were suggested to be related to the increase of strongly actin-bound states induced by OM since the central β-sheet is part of the transducer, the element of the motor domain that mediates the communication between the nucleotide and actin binding sites [25].

The lack of atomistic information on the OM-mediated modulation of myosin dynamics prompted us to perform Molecular Dynamics simulations on the cardiac motor domain bound to OM, where the effects of the drug on the dynamical properties of the protein are investigated for the first time with atomistic resolution. We find that OM has a double effect on myosin dynamics, inducing a) an increased coupling of the motions of the converter and lever arm subdomains to the rest of the protein, which produces a strong reduction in the amplitude of their motions and b) a rewiring of the network of dynamic correlations, which produces preferential communication pathways between the OM binding site and functional regions in the U50K subdomain. The residues and interactions mostly responsible for these effects are also identified and we discuss the possible use of these findings for the future development of improved drugs and the targeting of specific pathogenic mutations.

## Results

Molecular Dynamics (MD) simulations were performed on the motor domain of human cardiac myosin (cMotorD) starting from the X-ray structures of the Apo and OM-bound state [8]. Both structures were nucleotide-free. In addition to the motor domain, the simulated system also contained part of the lever arm helix (residues 769 to 783). In order to extend the exploration of the accessible conformational space and check for result reproducibility, four 300-ns simulations were run for each of the two binding states (Apo and OM-bound) using different initial coordinates for each simulation (A1, A2, B1 and B2 in S1 Table). These were generated by combining 1. two alternative conformations for the overall protein found in the X-ray structure (chains A and B), which represent the same myosin state in the actomyosin cycle (near-rigor state), but with subtle structural differences in the converter and lever arm region (Apo and OM-bound state) and in the transducer (OM-bound state only) [8] and 2. two alternative models for the loops with missing experimental coordinates (loop models 1 and 2, S1 Text). Differences between the Apo and OM-bound state were considered as reproducible if they were consistent across the different replicas, indicating that they were independent from the initial starting structure. The total simulated time amounted to 2.4 μs (2 X 4 X 300 ns).

In the following, we will first analyse the intrinsic dynamics of cMotorD in the Apo state, focusing on the behaviour of the converter and lever arm domains (collectively referred to in the following as CLD). We will then discuss the effect of OM binding on CLD dynamics, together with long-range effects on the rest of cMotorD.

### Uncoupled dynamics of the CLD in the unbound state of cMotorD

**Fig 2:**
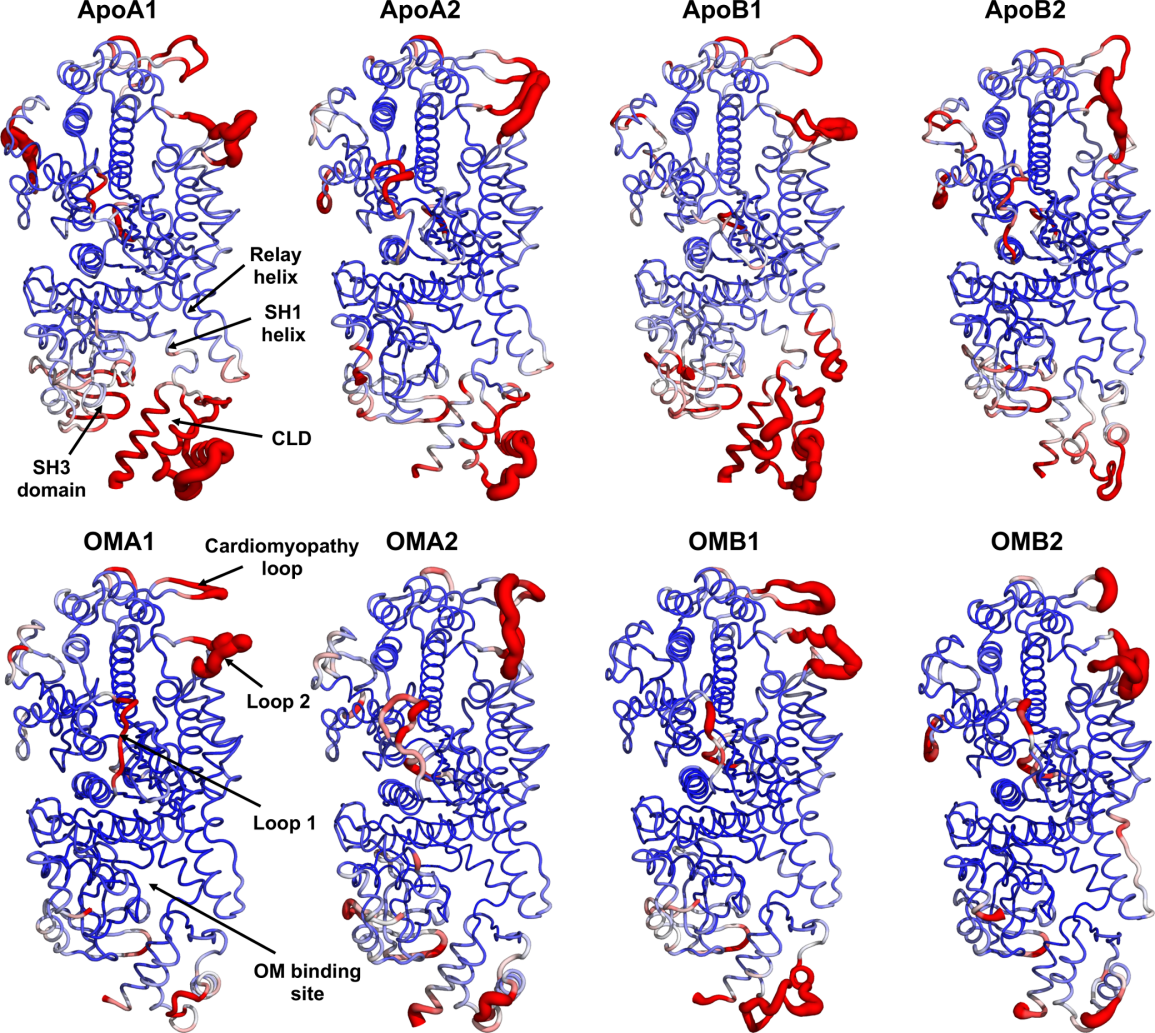
Mapping of C^α^ RMSF profiles onto the cMotorD structure. RMSF values from Apo and OM-bound simulations are colour mapped onto the cMotorD structure from blue (0 Å) to red (3.4 Å). The average structure is used for each simulation. The thickness of the tube representation is proportional to the RMSF value. High flexibility regions and the OM binding site are also labelled.

The overall dynamics of cMotorD during the Apo trajectories was first analysed in terms of the Root Mean Square Fluctuation (RMSF) of its C^α^ atoms. A high flexibility was found for the long loops in the U50K (loop1 and cardiomyopathy loop) and L50K (loop2) subdomains (Fig 2), as expected on the basis of their length and of the disordered nature of loops 1 and 2 [26]. Interestingly, large RMSF values were observed for the CLD and, to a less extent, the nearby SH3 domain and relay helix. These large amplitude motions of the CLD domain were found in all the Apo trajectories, albeit with a reduced extent for ApoB2, indicating that they are independent from either the overall initial conformation of the domain (chain A or B) or the loop model used (1 or 2).

**Fig 3:**
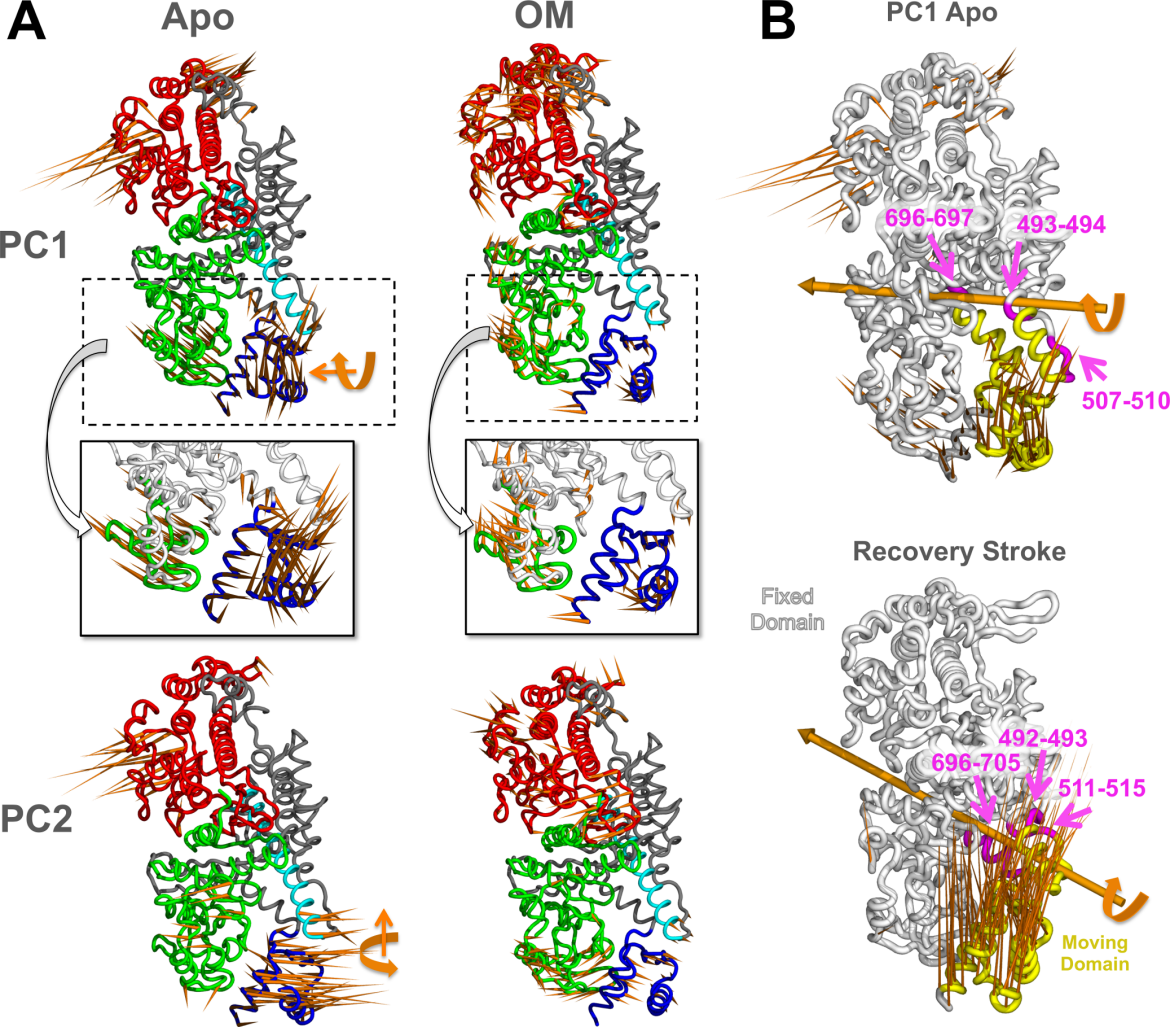
Collective motions in Apo and OM-bound simulations. A. Porcupine representation of PC1 (top panels) and PC2 (bottom) in ApoA1 (left panels) and OMA1 (right) simulations. The orange spikes show the direction and relative amplitude of motion of each residue along the PC. The approximate direction of the CLD hinge axis is also shown for Apo simulations (orange arrows). The two insets show the anti-correlated (Apo) and correlated (OM-bound) motions of the CLD (blue) and SH3 (green) subdomains. B. DynDom dynamic domain decomposition for ApoA1 PC1 (top) and the recovery stroke (bottom). The analysis was performed on the structures with minimum and maximum PC1 value from the MD simulation and on the experimental structures representing the pre-power stroke (PDB ID: 1QVI) and near-rigor (PDB ID: 1SR6) states for the recovery stroke. The fixed (white) and moving (yellow) domains identified by DynDom are shown, together with the hinge axis (orange) and the hinge regions (magenta).

The cMotorD dynamics was further investigated by identifying the collective motions with a Principal Component Analysis (PCA). PCA was performed on the C^α^ atoms only and by excluding the modelled loops to reduce the noise from these disordered regions (Methods). The first two principal components (PC1 and PC2) of each trajectory, describing the most important collective motions observed during the simulation, accounted together for a significant portion of the overall variance (S2 Table), ranging from 33% (ApoB2) to 65% (ApoB1). Two recurring types of motions were found, involving mainly quasi-rigid rotations of the CLD around two different hinge axes, with hinge regions located in the SH1 helix and the relay helix/loop (Fig 3A and 3B). These CLD motions were the main contributions to the PCs in almost all the cases, even if in different proportions, so that each type of rotation was found either in PC1 or in PC2 according to the specific simulation (S1 Fig). The exceptions were PC2 from ApoA2 and PC1 from ApoB2, where rotational motions of the upper 50K domain were found to be of comparable or larger amplitude than the CLD ones.

**Fig 4:**
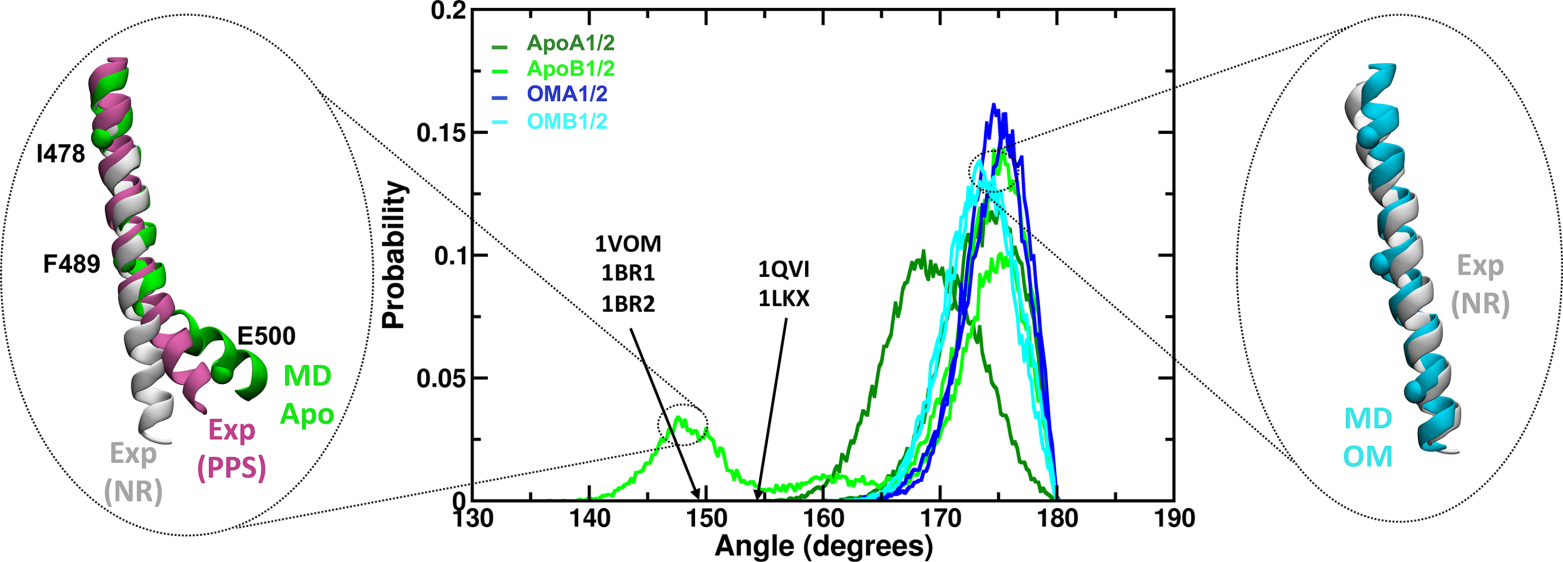
Bending of the relay helix during the MD simulations. The relay helix bending was measured by calculating the angle formed by residues I478, F489 and E500 (C^α^ atoms only). The central plot shows the probability distribution of the angle values observed during Apo (green hues) and OM-bound (blue hues) simulations. Arrows indicate the approximate value of the angle measured for representative experimental structures of the pre-power stroke state (PDB IDs shown). Apo trajectories showed a higher propensity for bent conformations (lower angles) than OM-bound simulations. The insets show representative Apo bent (green, left) and OM-bound straight (cyan, right) structures. Experimental structures of the relay helix in the near-rigor (NR, white, PDB ID: 1SR6) and pre-power stroke (PPS, magenta, PDB ID: 1QVI) state from scallop myosin are also represented as reference.

It is interesting to note that the CLD rotation described by PC1 (ApoA1) has similarities with the conformational changes occurring in the same region during the acto-myosin cycle. In particular, the transition from the post-rigor to pre-powerstroke state (‘recovery stroke’) as described by X-ray structures involves a rotation of the CLD around an axis with similar direction as PC1 and similar hinge regions (Fig 3B), but with an amplitude much larger than the one observed here (~70° instead of ~ 30° in ApoA1). The CLD rotation in the recovery stroke is also known to be associated with the formation of a kink in the relay helix [6]. Remarkably, a significant bending of the relay helix was found during the ApoA1 and B1 simulations (Fig 4). In particular, at the end of the ApoB1 simulation the helix formed a kink similar in amplitude and position to that found in the X-ray structures of the pre-powerstroke state (Fig 4, left inset). The bent part of the helix was found to adopt different orientations in addition to those observed in the experimental structures. Overall, these data suggest that the motor domain can partially sample motions similar to those involved in the recovery stroke even in the absence of ATP.

To summarise this section, we found that the intrinsic dynamics of cMotorD in the Apo state is dominated by the CLD, which is relatively free to move as a quasi-rigid domain. These types of CLD motions, namely rigid rotations around hinges located in the SH1 and relay helices, are consistent with the changes associated with the actomyosin cycle, even if of smaller amplitude. Moreover, in two of the simulations the CLD motions were associated with a significant bending of the relay helix.

### OM strongly couples the CLD to the rest of cMotorD

**Fig 5:**
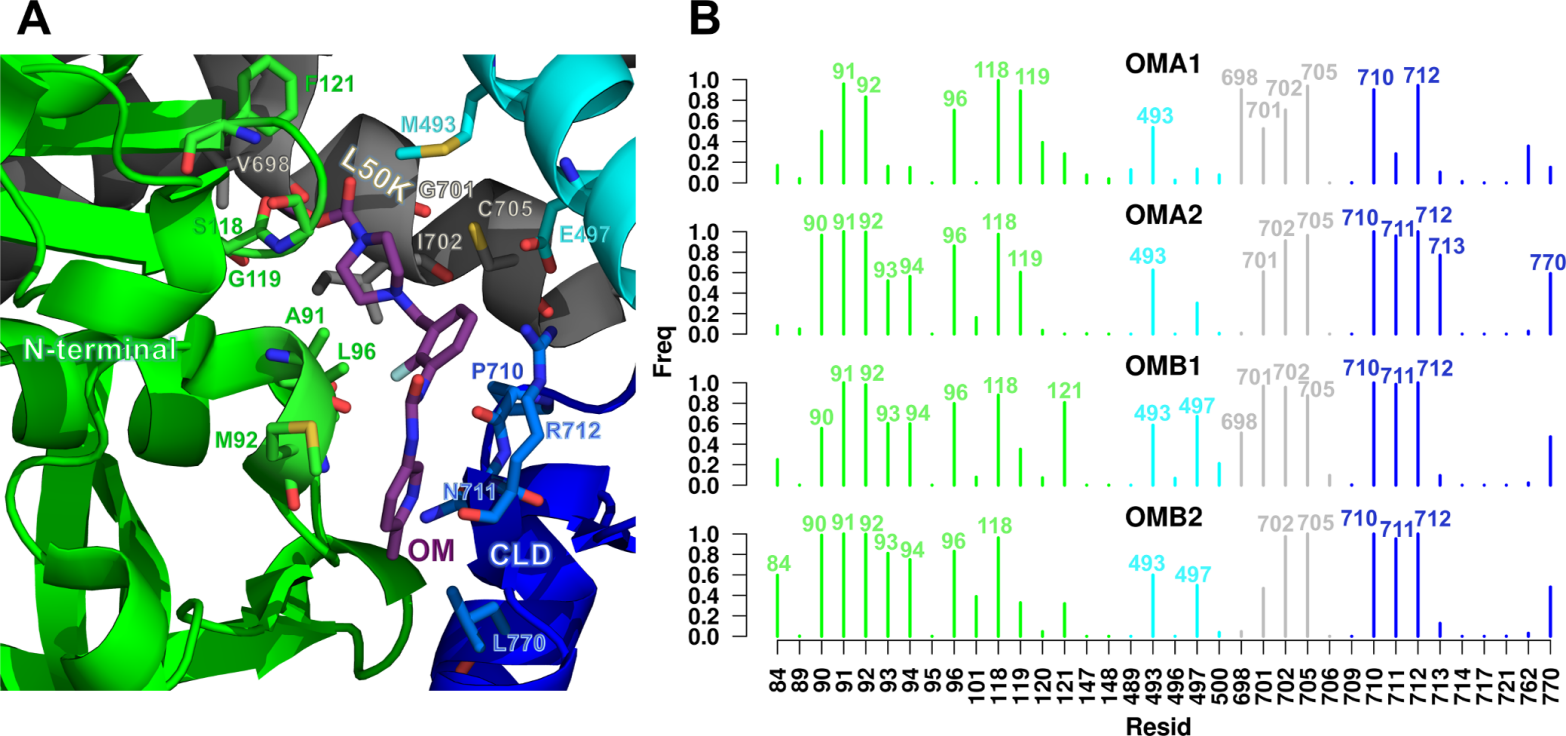
OM-protein interactions. A. X-ray structure of the OM-binding site in cardiac myosin (PDB ID: 4PA0, chain A). Residues within contact distance from OM are shown as sticks and coloured according to the subdomain they belong to. B. Frequency of OM-residue contacts during OM-bound MD simulations. Each bar represents the fraction of the simulated time for which the corresponding residue was found in contact with OM (minimum distance between any non-hydrogen atom < 4 Å). Residues are labelled for frequencies larger than 0.5.

As mentioned above, OM binds to a critical region of cMotorD where it can interact with multiple subdomains at the same time (Fig 5A). Indeed, the X-ray structures show that in both chain A and chain B conformations OM is in contact with residues of the N-terminal domain (A91, M92, L96, S118, G119, F121), the relay helix (M493, E497), the SH1 helix in the L50K subdomain (V698, G701, I702, C705) and the CLD (P710, N711, R712, L770)[8].

OM was stably bound to the protein during the simulations (S2A Fig). Its interactions with M90, A91, M92, L96, S118, M493, I702, C705, P710 and R712 were found to be particularly stable, since the OM-residue distance was below 4 Å for at least 50% of the frames in all the OM-bound trajectories (Fig 5B and S3 Table). Chain A and chain B simulations produced similar contact fingerprints. Residues A91, S118, L120, C705, N711, R712 and K762 formed transient hydrogen bonding interactions with OM, which were dependent on the specific conformation adopted by OM (S4 Table and S3 Fig), while strong hydrophobic interactions with M90, A91, M92, L96, M493 and I702 were observed in all the simulations (side chains of non-polar residues within 4 Å from OM for at least 50% frames, S3 Table). The molecule was relatively flexible (S2B Fig), as expected from the fact that it adopts different conformations in the X-ray structures of chain A and chain B (blue and light blue sticks in S2C Fig). A cluster analysis (S1 Text) performed on the concatenated trajectories (S5 Table and S2C Fig) showed that the most populated cluster (82%) was sampled almost equally in all the simulations (except for OMA1, where it contributes with a smaller proportion), with the representative structure having an RMSD from the X-ray structures of 2.2 (chain A) and 2.1 (chain B) Å (red sticks in S2C Fig, left panel). A significantly different conformation (cluster 4, S5 Table) was sampled for a short amount of time (2%) at the end of the OMA1 simulation, where the methyl-pyridinyl ring was shifted upwards toward the β1-β2 loop (S2C Fig, right panel). This conformation might be related to the restructuring of the OM binding pocket recently observed in the pre-powerstroke state, where the pocket is shifted upwards as a result of the lever arm motion during the recovery stroke [27].

**Fig 6:**
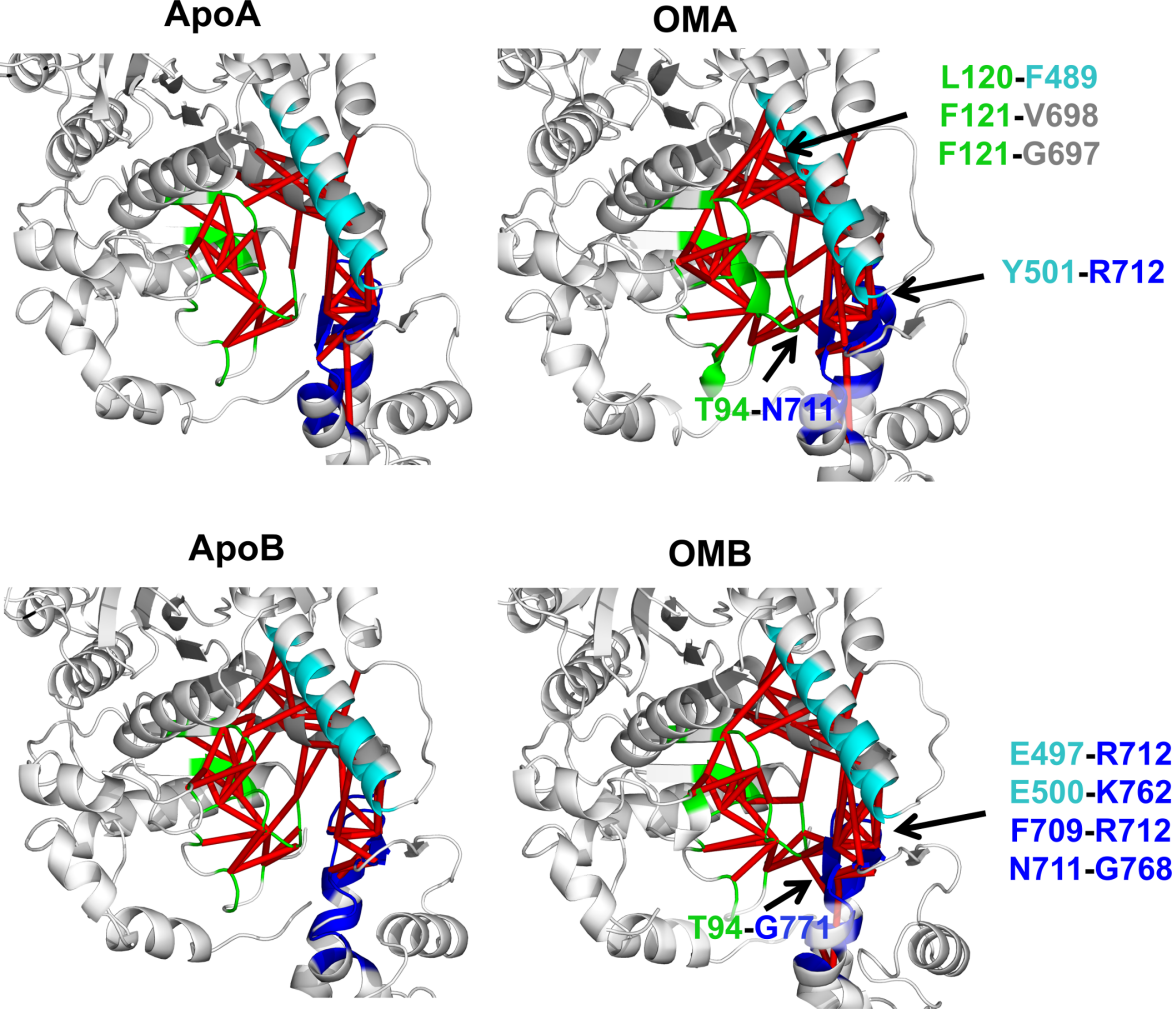
Network of inter-residue contacts in the OM-binding site. Red edges connect pairs of residues that are found in contact for at least 70% of the simulation. All the residues within 8 Å from OM were included in the analysis. Contacts are reported for replica 1 only (see S4 Fig for all the replicas). For each chain, pairs that were consistently found in contact in both OM-bound simulations and in none of the Apo ones are labelled.

The behaviour of the OM-binding site was further analysed by monitoring the inter-residue contacts in proximity of OM and comparing their stability with that found in the Apo trajectories (Fig 6 and S4 Fig). OM-bound simulations in general presented a larger number of stable inter-residue contacts (inter-residue distance < 4 Å for at least 70% of the simulation). OM-stabilised contacts were found between the N-terminal domain and the converter (T94-N711 for OMA simulations and T94-G771 for OMB), and the relay helix and the converter (Y501-R712 for OMA and E497-R712 and E500-K762 for OMB). Moreover, OMA trajectories showed additional hydrophobic contacts between the central β-sheet and the SH1 helix (F121-V698 and F121-G697) and the β-sheet and the relay helix (L120-F489), while stronger intra-CLD contacts (F709-R712 and N711-G768) were found in OMB trajectories. The reduced number of CLD OM-stabilised contacts found in chain A simulations is probably due to the small re-organisation of CLD observed in these trajectories. Indeed, chain A and B initial structures have small differences in the orientation of the CLD, with the chain B CLD slightly rotated in the direction of the recovery stroke (S5 Fig). During the OM-bound simulations, chain A relaxed towards the chain B conformation, while the chain B conformation was stable throughout the whole trajectory (S6 Fig).

The overall effect of OM on cMotorD interactions was then to enhance the contacts between the different subdomains that compose its binding site, both by directly interacting with them and by stabilising the inter-residue contacts around it. This had a dramatic effect on the overall flexibility of the protein, as shown by the RMSF profiles (lower panels in Fig 2 and S7 Fig). Indeed, a significant reduction in mobility was found for the CLD, together with the neighbouring SH3 domain and the SH1 and relay helices. Correspondingly, the first two collective motions (PC1 and 2), while showing a higher diversity across the OM-bound replicas compared to the Apo simulations, were consistently found to have higher contributions from the other subdomains (the upper domain for chain A simulations and either the N-terminal domain or the lower domain for chain B) rather than the CLD (Fig 3A and S8 Fig). Moreover, when the CLD contributed significantly to the PCs, its motion was correlated with the neighbouring subdomains rather than anti-correlated (insets in Fig 3A). The overall amplitude of the global motions was also significantly reduced compared to the Apo simulations (S9 Fig). The results obtained comparing the single simulations were confirmed by a PCA performed on the combined Apo and OM-bound trajectories (S10 Fig). The directions in the conformational space that best discriminate between the two binding states (43% of the overall variance) are represented by the two types of CLD hinged rotations observed in the single Apo simulations (S10A Fig). A projection onto PC1 and 2 shows a clear separation of the Apo and OM simulations, since the Apo trajectories span a much larger range of values along both components while the OM trajectories are located in the same region of the space (S10B Fig). Finally, no significant bending was found for the relay helix in the presence of OM (blue hues and right inset in Fig 4). A comparison of representative structures from Apo and OM-bound trajectories is presented in S11 Fig, where it is possible to see the different arrangement adopted by the CLD and the relay helix at the end of the simulations.

**Fig 7:**
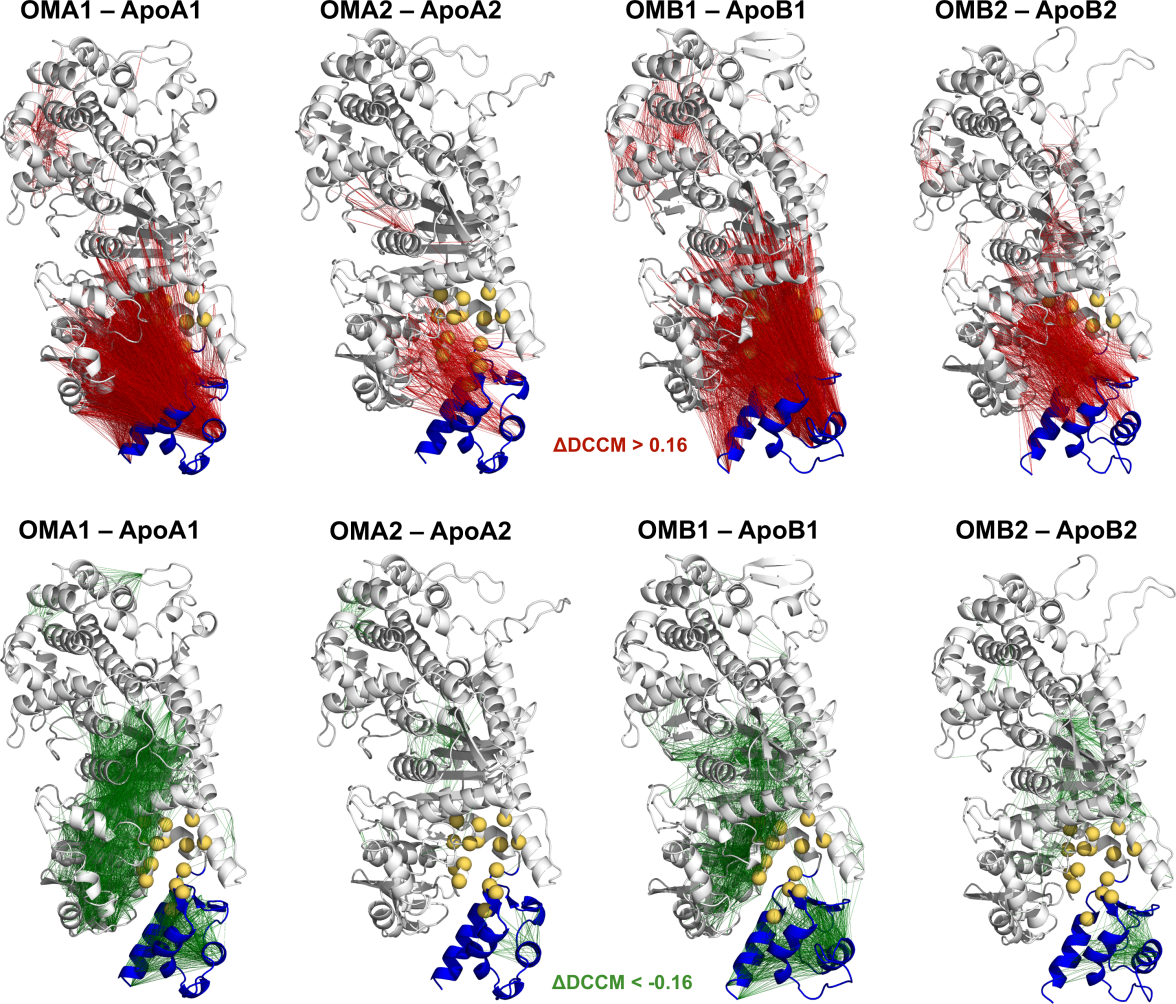
Comparison of dynamic cross-correlation networks. Difference DCCM matrices (ΔDCCM) calculated between pairs of OM-bound and Apo simulations are mapped onto the initial structures of each OM-bound simulation. Edges connect residue pairs that have a positive (red) and a negative (green) ΔDCCM value, using a threshold of 0.16. Residues in the OM-binding site are represented as yellow spheres, while the CLD domain is coloured in blue.

The previous analysis indicates that the CLD motions found in the Apo simulations are significantly decreased in the presence of OM and that CLD moves in a concerted way with other subdomains rather than freely rotating around the SH1 hinge as was instead observed in the unbound state. In agreement with this, the comparison of the dynamical cross-correlation matrices (DCCM) showed that OM binding induces in all the simulations an increase of the correlations between the CLD and the rest of the protein (red lines in Fig 7 and red dots in S12 Fig), in particular the N-terminal domain, while at the same time decreasing the CLD intra-domain correlations (green lines in Fig 7 and green dots in S12 Fig).

### OM rewires the network of local correlations within cMotorD

The OM-induced changes in the dynamical correlation within cMotorD were further analysed by reconstructing the network of local correlated motions using the M32K25 Structural Alphabet (SA) (Methods). This type of analysis highlights correlations between changes in the conformational state of 4-residue fragments of the protein backbone during an MD simulation. While the PCs and the DCCM networks presented in the previous sections are usually dominated by hinge motions of quasi-rigid dynamical domains, local correlations represent more subtle effects involving correlated changes in the local shape of the protein backbone.

**Fig 8:**
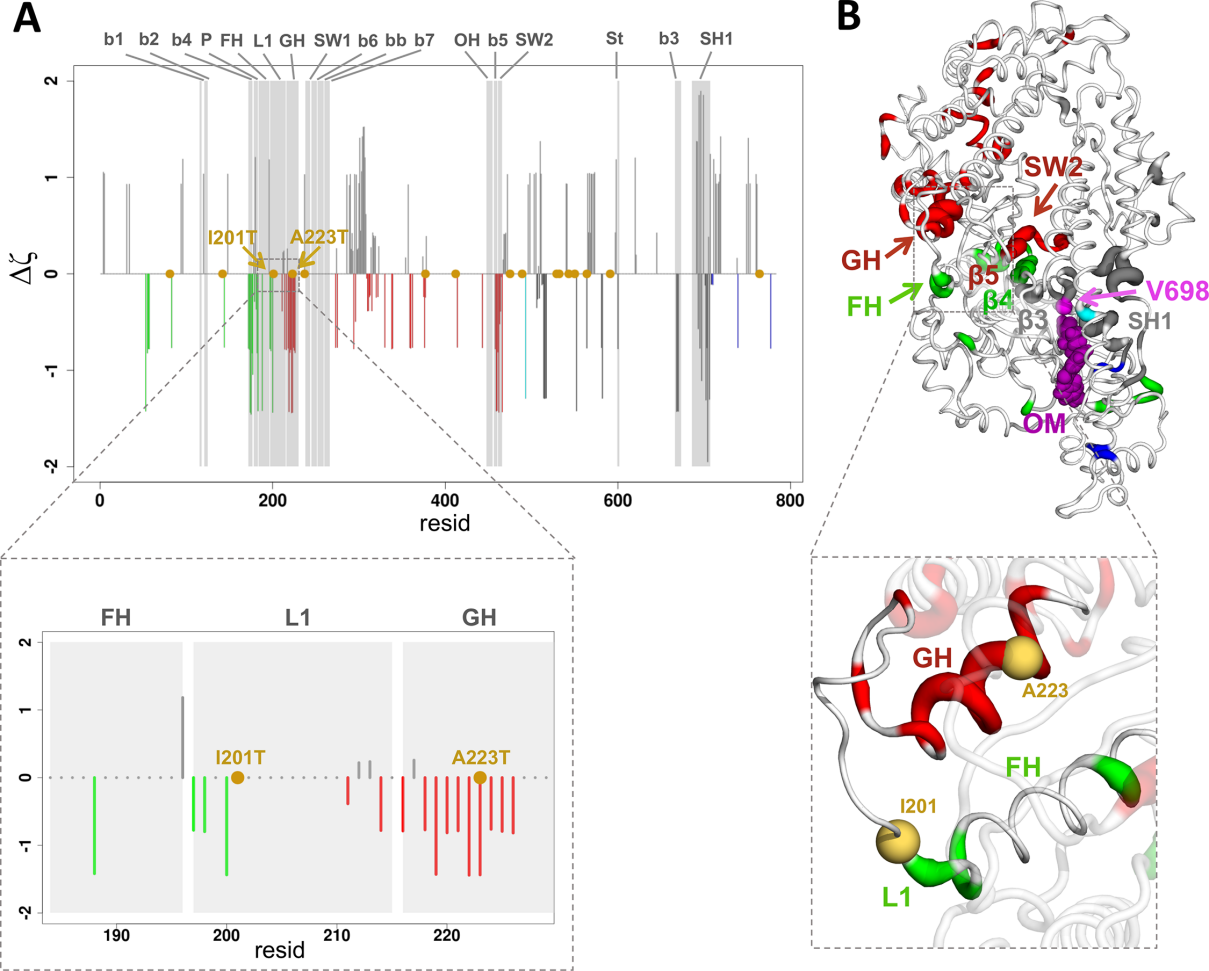
Preferential connections in the local correlation network between the OM-binding site and functional regions in cMotorD. A. Plot of the difference between OM and Apo preferential connection scores (Δζ), calculated using fragment V698 as source site. The scores are derived from a consensus correlation matrix calculated over the four simulations of each binding state. A negative value indicates a stronger preferential connection to V698 in OM-bound simulations compared to Apo ones. Negative bars are coloured according to the subdomain. The position of DCM-related mutations is indicated with a yellow dot. Grey areas highlight the position of functional regions, namely the seven strands of the central β-sheet (b1 to b7), the P-loop (P), the F helix (FH), Loop 1 (L1), the G helix (GH), Switch 1 (SW1), the β-bulge (bb), the OH loop (OH), Switch 2 (SW2), the Strut (St) and the SH1 helix (SH1). The inset shows a close-up view of the fragments in the F-helix, the loop L1 and the G-helix (resid 185–228). B. Mapping of the Δζ scores onto the initial structures of OMA1. Only regions with negative values are coloured. The thickness of the tube is proportional to the magnitude of Δζ values. A close-up view of the F-helix, the loop L1 and the G-helix is shown in the inset, with the position of residues I201 and A223 indicated by yellow spheres.

Local correlation networks were calculated for each simulation of the Apo and OM-bound state. A consensus network was then generated for each binding state (S1 Text), resulting in two networks to be compared (Apo and OM). The networks were first analysed by calculating a preferential connection score ζ between each 4-residue fragment in the OM binding site and the rest of the cMotorD domain (Methods). Fragments with negative ζ values have a network distance from OM-binding fragments that is smaller than the average, so that they can be considered as preferentially connected to the OM-binding site. The scores obtained from the OM-bound simulations were then compared with the Apo ones and Δζ differences were calculated by subtracting the Apo from the OM-bound profiles (Fig 8). In the following, we will focus on the fragment starting with V698 (or fragment V698), but consistent results were obtained for the other OM-binding site fragments (S13 Fig).

The ζ values in most of the functional regions show either no change or a decrease upon OM binding (Δζ < 0), while increased values (Δζ > 0) were usually observed outside these regions (Fig 8A). This indicates that the OM-binding site tends to have a stronger preferential connection to the functional regions in OM-bound simulations compared to the Apo ones. Indeed, the Apo simulations showed either a reduced (smaller |ζ| values compared to the OM ones) or no preferential connection (ζ = 0) to these regions (S14 Fig). The most pronounced increases in preferential connection were observed for the G helix, the β5 strand and Switch 2 in the ATP binding site (Fig 8B), which consistently showed negative Δζ values for all the OM-binding site fragments (S13 Fig). Increased preferential connections were also observed for the β3 and β4 strands and part of Loop 1 in most of the cases. Interestingly, mapping the mutations known to be associated with dilated cardiomyopathy (DCM) [28] onto the Δζ profiles (yellow points in Fig 8), shows that some of them are in regions preferentially connected to OM. In particular, I201T (Loop 1) and A223T (G helix) are found in functional regions with consistently negative Δζ values. This would suggest that their effect could potentially be counteracted by OM binding.

**Fig 9:**
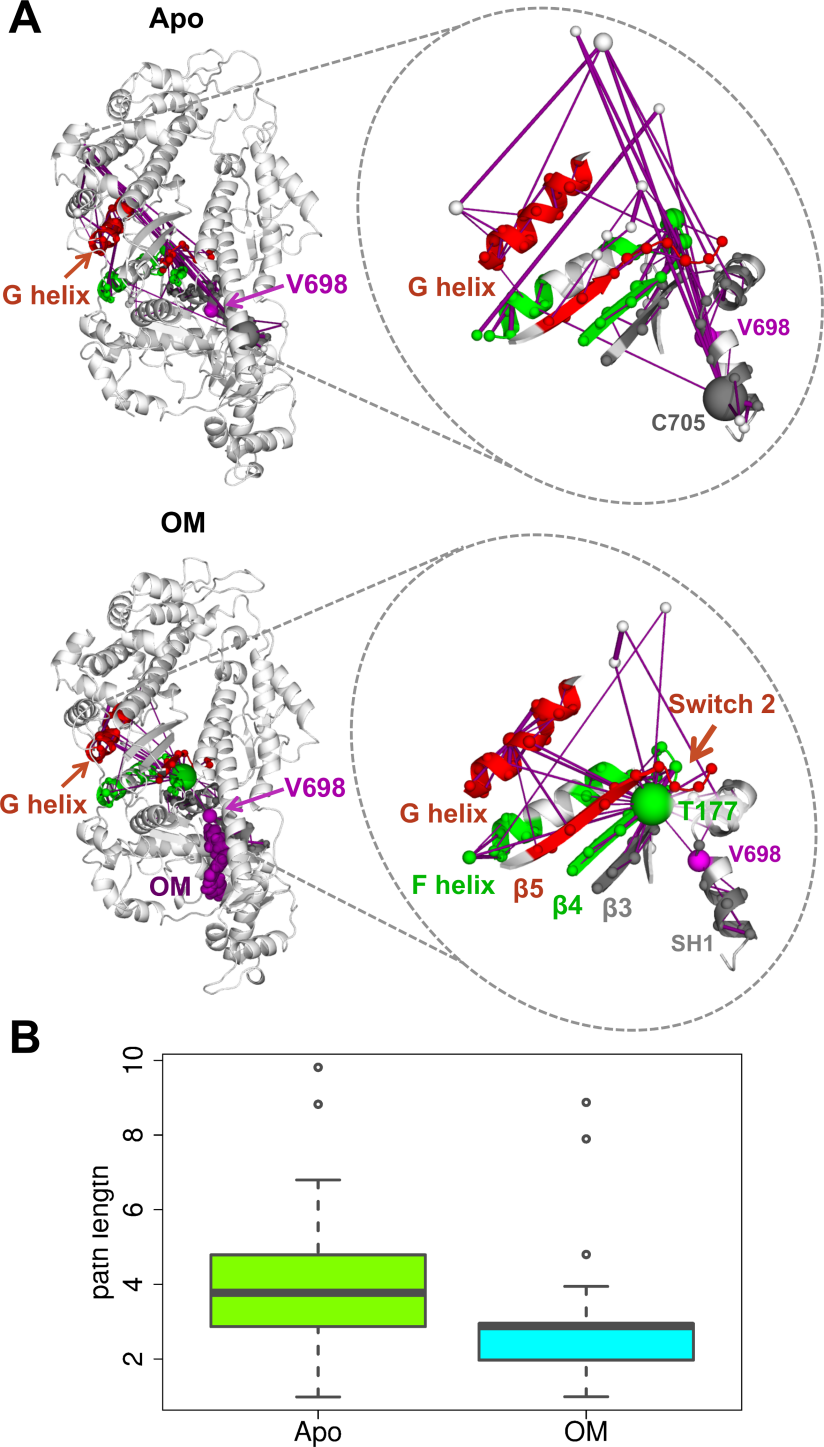
Communication pathways from the OM-binding site in the network of local dynamic correlations. A. The shortest paths calculated from fragment V698 (magenta) to selected functional regions are reported as purple edges for Apo (top) and OM-bound (bottom) simulations. Fragments are mapped onto the structure by using the first residue in the fragment. The functional regions (F and G helix, β3–5, Switch 2 and SH1 helix) were selected among those with a stronger preferential connection to V698 in OM-bound simulations compared to the Apo ones. The nodes in the paths are represented as spheres coloured according to the subdomain they belong to and with a radius proportional to the number of paths going through them (so that larger spheres correspond to hubs). Edge thickness is proportional to the correlation value. B. Boxplot representation of the path lengths for the shortest paths reported in A for Apo (green) and OM-bound (cyan) simulations.

The ζ score changes induced by OM in regions related to myosin function suggest the presence of significant differences in the local correlation network of OM-bound and Apo states. To investigate this further, the shortest paths were determined between fragment V698 and the preferentially connected functional regions identified above (Methods). The comparison of the OM-bound and Apo results (Fig 9A) shows that the endpoints are more directly connected in the OM-bound network (bottom panels) than the Apo one (top panels). As expected, the paths connecting the OM-binding site (V698, magenta) on one side and the functional regions (coloured cartoon) on the other involve a smaller number of edges (purple lines) and are thus shorter in terms of distance in the network (Fig 9B) in the OM-bound simulations. The network representation also shows that the paths in the Apo networks involve more nodes that are distant in space from the end points compared to the OM-bound one (Fig 9A). Moreover, both networks contain a hub node with a large number of connections (fragment T177 for the OM-bound network and fragment C705 for the Apo one), but while T177 is directly connected to most of the functional regions, C705 needs to go through other nodes to reach the endpoints.

**Fig 10:**
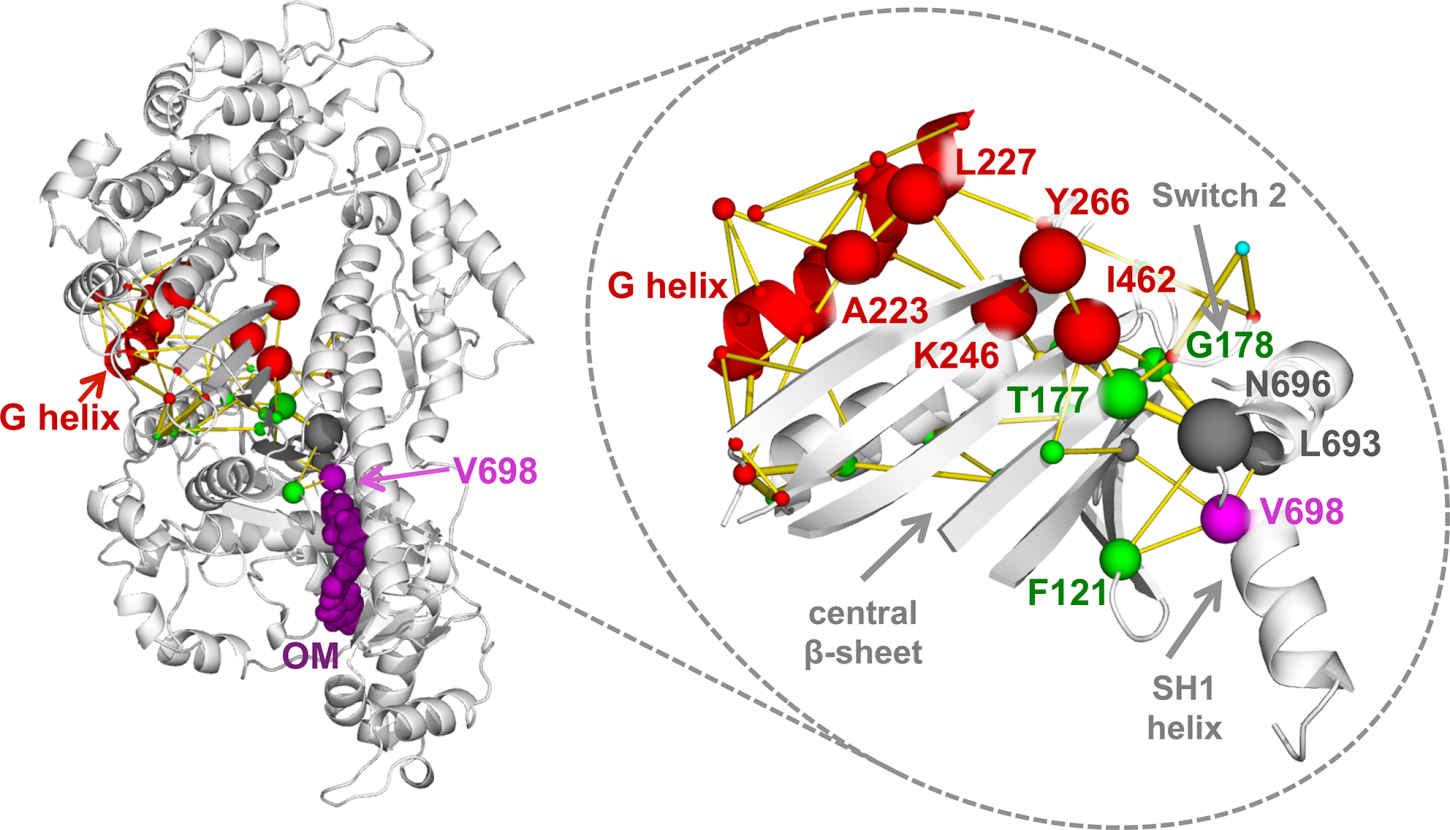
Pathways connecting the OM-binding site and the G-helix in the contact change network. The shortest paths connecting V698 (magenta) and G helix residues (red) in the network of OM-Apo contact changes are represented as yellow edges. The paths are calculated from a consensus contact change matrix calculated over all the simulations. Only contacts with a change in frequency larger than 0.1 were considered. The edge thickness is proportional to the difference between OM and Apo contact frequency. For each pair of endpoints, the top 5 shortest paths are represented. The nodes in the paths are represented as spheres coloured according to the subdomain they belong to and with a radius proportional to the number of paths going through them. The top 10 residues for number of paths are also labelled.

In order to identify the residues and interactions mostly involved in this reorganisation of the local correlation network, we analysed the modifications in inter-residue contacts induced by OM binding (i.e. contacts stabilised or destabilised by OM) in the region between the OM-binding site and the G-helix (Fig 10). Contact matrices were first determined for each Apo and OM-bound simulation by calculating the fraction of the trajectory for which each residue pair was found in contact. A consensus contact matrix was then derived for each binding state and the OM-Apo difference was used to obtain a matrix representing a network of contact changes (S1 Text). Calculating the paths connecting V698 (magenta sphere) and the G-helix residues (red cartoon) in the network (yellow edges) shows chains of contact changes going through the two sides of the central β sheet. The side closer to Switch 2 contains the nodes with the largest number of paths going through them (larger spheres), suggesting that the corresponding residues (namely F121, N696, L693, T177, G178, I462, K246, Y266, L277 and A223) are important in mediating the effects on the local correlation network induced by OM binding. Interestingly, residues T177 and G178, which are part of the hub fragment T177 in the OM-bound local correlation network (Fig 9A), are involved in most of the paths (83% of the paths contain either T177 or G178, S6 Table) and they participate in contacts that have among the largest changes in frequency upon OM binding (S7 Table).

## Discussion

In this work, we used MD simulations to investigate the effect of the sarcomeric modulator omecamtiv mecarbil (OM) on cardiac myosin dynamics with atomistic resolution. Simulations were performed to reconstruct the sub-microsecond dynamics of the motor domain of cardiac myosin (cMotorD) in the absence and presence of OM, starting from the recently solved structures of the Apo and OM-bound cMotorD in the near-rigor state[8]. The light-chain containing regulatory domain (RD) was not considered here since no experimental structure is currently available for the cardiac isoform, however the simulated system included a small fragment of the lever arm helix. The regulatory role of the RD in cardiac myosin is mediated by the phosphorylation of a disordered portion of the regulatory light chain (RLC), which is thought to be involved in the regulation of the transition from an inactive (off) to an active (on) form of the two-headed myosin molecule in the thick filament [29, 30]. OM has been shown to leave the RLC phosphorylation levels unchanged [31], suggesting that the molecular mechanisms mediated by OM are independent from those mediated by the RD.

To the best of our knowledge, the present simulations represent the first fully atomistic simulations of cardiac myosin bound to a small molecule modulator. Previous computational works have focused so far on the effect of nucleotide [32] or actin binding [33] on myosin dynamics, the interactions between actin and myosin [34], the release of Pi [35], the modelling of the recovery stroke [36–39] or in general of the coupling between the actin binding site, the nucleotide binding site and the converter [40–43]. A significant part of these studies used enhanced sampling techniques to accelerate the transitions between the different states in the actomyosin cycle [35, 36, 38–41, 43, 44], while unbiased simulations with length > = 50 ns have been performed only recently [32, 33, 37, 45] thanks to the increase of the available computational power.

The simulations presented here show that, in the absence of OM, the dynamics of cMotorD is dominated by the hinge motions of the converter+lever arm helix subdomain (CLD). These have some resemblance to the CLD rotation observed during the transition from the experimental near-rigor to pre-powerstroke structure (recovery stroke) in that a) they involve similar hinge regions and b) they are associated with the bending of the relay helix. However, the CLD motions in the simulations seem to be more heterogeneous since, in addition to recovery stroke-like rotations (Fig 3B), the domain can perform rotations in other directions (e.g. Apo PC2 in Fig 3A). Moreover, the amplitude of the rotations is much smaller than in the recovery stroke, which is expected on the basis of the length of the simulation and the absence of ATP. The motions observed here, while not representing an actual transition to the pre-powerstroke state and while they might have been enhanced by the absence of the Regulatory Domain, suggest a conformational selection scenario for the recovery stroke, where the type of motions involved in the stroke are already partially sampled by the CLD before the binding of ATP. This finding is in agreement with the emerging model that CLD rotation is stabilised by the closure of Switch 2 upon ATP binding rather than being induced by it, so that it can occur at least partially before the changes in the nucleotide binding site take place [12, 43, 46]. This would also explain the recent observation that myosin can be trapped by inhibitors in intermediate states of the recovery stroke without closure of Switch 2 [12]. Moreover, a decoupling between Switch 2 and lever arm motions is consistent with the recent observation of a new structural state of Myosin VI where a large conformational change of Switch 2 does not produce a corresponding change in the lever arm position [47].

OM is shown by the simulations to have a double effect on myosin dynamics. The first is to dramatically reduce the CLD motions observed in the Apo state and couple them to the rest of cMotorD, as indicated by the large decrease in CLD motions and the increase of positive correlation between the CLD and the other regions, in particular the SH3 subdomain in the N-terminal region. A possible role of this subdomain in myosin activation is also suggested by the recent observation that one of the few other myosin activators currently known might bind to SH3[48]. Our results indicate that OM acts as a “glue” between the different subdomains that compose its binding site, both by directly interacting with them and by stabilising pre-existing inter-domain interactions. This effect was observed albeit with different extent in all the simulations, indicating that it is independent from the specific starting conformation.

The second OM-induced effect emerging from the simulations is a reorganisation of the network of correlated local motions, which results in a more efficient and direct connection between the OM-binding site and functional regions compared to the Apo state. In particular, the networks show the formation of preferential pathways between the OM binding site and distant U50K regions close to ATP binding site, namely the G helix and Switch 2. The communication between sites is mediated by a chain of OM-induced contact changes involving residues in the central β-sheet. Preferential connections in the dynamic correlation networks have been previously observed to be involved in allosteric communication [49].

Our results thus indicate that OM can modulate the cMotorD dynamics through at least two different molecular mechanisms, which would explain its complex and apparently contradictory effects on the kinetic parameters of the actomyosin cycle[16]. In particular, assuming that OM can induce similar effects on the pre-powerstroke state, the reduction of CLD rotational motions upon OM binding might explain the strong reduction in the powerstroke rotation rate measured with FRET [16] and the overall decrease of the actin sliding velocity [14, 15]. This inhibitory effect on the lever arm motions is considered to be consistent with the overall increase in muscle contractility produced by OM binding, since it increases the fraction of time spent by myosin in the force generating state where it is strongly bound to actin [14, 16]. On the other hand, the enhanced correlation with the G helix and Switch 2 might be related to the change in the number of myosin molecules strongly bound to actin. Indeed, the G helix has been shown to move concertedly with Switch 1 during the opening of the actin binding site when myosin dissociates from actin [40]. Changes in the conformation and/or dynamics of the G helix could affect the energetics of the reverse process, where strongly bound myosin states are produced upon closure of the actin binding site. Moreover, a Pi release mechanism involving mainly Switch 2 rather than Switch 1 motions has been recently suggested on the basis of a newly found structural state of Myosin VI [47].

During the revision process of this paper, a new OM-bound structure has become available, where OM is bound to the pre-power stroke state [27]. Simulations using the same protocol will confirm whether OM has similar effects on the dynamics of this state, which is further down the actomyosin cycle. Moreover, simulating the transition between the near-rigor state studied in this work and the pre-power stroke state will allow the quantification of possible effects on the energy landscape of the recovery stroke, in particular on the relative stability of the two states and the energy barrier between them [50].

Our findings suggest that the future development of OM-based drugs could act on two different sets of molecular features to produce novel compounds with improved action. In particular, changes in the interactions with the central β-sheet could modify the strength of the preferential connection to regions close to the ATP binding site and ultimately the transition to strongly bound states. On the other hand, modifications in the interactions with the N-terminal domain or the CLD could change the degree of coupling between the CLD and the rest of cMotorD and thus its rate of rotation during the powerstroke. Obtaining an optimal balance between these two effects could be essential to produce drugs with reduced side effects or specific therapeutic properties.

The present results can also be used to identify specific pathogenic mutations to be targeted by OM. Indeed, small molecule activators of myosin have been previously proposed as possible drugs to counteract the effect of dilated cardiomyopathy (DCM) mutations, since these are usually associated with a decrease of the power output of the cardiac muscle [4]. Comparing the distribution of the currently known DCM mutations [28] with the regions preferentially connected to the OM-binding site can give information on the mutants that are most likely to be directly affected by OM binding (yellow dots in Fig 8). Mutations I201T [51] (Loop1) and A223T [52] (G helix) are particularly interesting since a) they are located in regions with high preferential connection and b) they are far from the OM binding site, so they are less likely to directly interfere with the drug binding. Further investigations on OM-bound mutants will be able to highlight if OM has a rescuing effect on them, namely if it can reverse any effect induced by these mutations on the structural and dynamical properties of myosin.

## Methods

### System preparation

The initial structure of the motor domain of the human β-cardiac myosin (residues 1 to 783 of UniProt sequence P12883) was extracted from the Protein Data Bank (PDB) for the OM-bound (PDB ID: 4PA0) and Apo (PDB ID: 4P7H) state[8]. Both structures are nucleotide-free. Two chains (A and B) were found in both PDB files, which show structural differences in the converter, lever arm and central β-sheet. Simulations were started from both chains and labelled accordingly (S1 Table).

Homology modelling was used to model unsolved parts of the protein in the X-ray structure, which included loops localised either in the actin-binding region or in the converter (S8 Table). MODELLER 9.15 [53] was used to model the missing loops by keeping the rest of the structure unchanged, using as templates structures from chicken skeletal (PDB ID: 1M8Q) and smooth (PDB ID: 1BR1) muscle myosin. Since these loops were expected to be highly flexible, to make sure that the results obtained from the simulations were not dependent on their specific initial structure, two different initial loop conformations were generated. These were then used as starting points to run two different replicas for each system (*x* = 1 or 2 in S1 Table), resulting in a total number of eight different MD simulations (further details on loop modelling can be found in S1 Text).

### MD simulation setup and protocol

The protein was described using the Amber99SB*-ILDN force field, which has been extensively tested for its ability to reproduce the correct relative stability of secondary structure elements and side-chain conformations in proteins [54]. For compatibility with the force field used for the protein, the OM ligand was described using parameters from the General Amber Force Field (GAFF) [55]. PyMOL (version 1.8.2.0) [56] was used to add the hydrogen atoms to the OM structure and generate the connectivity table. The Antechamber and tLeap tools from the AmberTools 15 [57] suite were used to generate the files with the OM topology and force field parameters, together with the initial coordinates. These were then converted to GROMACS format using Acpype[58]. The atomic point charges were generated using the AM1-BCC[59] method using either the chain-A or the chain-B conformation of OM. As expected, only small differences were found in the two sets of charges, with an overall RMSD of 0.003 a.u. The atom types and charges used for OM are reported in S9 Table, while OM structural formula is reported in S15 Fig together with the atom numbering. Additional details on OM parametrisation are described in S1 Text and S10 Table.

All MD simulations were performed using GROMACS 4.6.7 [60]. Each system was solvated using a truncated octahedral box of TIP3P water molecules. Periodic boundary conditions were applied, using the Particle Mesh Ewald (PME) method for electrostatic interactions, a 9-Å cutoff for the direct space sums and for van der Waals interactions, and long-range corrections to the dispersion energy. Energy minimisation of each system was followed by equilibration first in NVT (T = 300 K) and then in NPT conditions (T = 300 K, p = 1 bar) for a total 6.5 ns. Production NPT runs were then performed for 300 ns, saving the coordinates every 1 ps. The stability of the simulations was checked by monitoring the Root Mean Square Deviation (RMSD) from the initial structure (S16 Fig) and the time evolution of the DSSP secondary structure annotation (S17 Fig). The detailed MD protocol with full references can be found in S1 Text.

### Analysis of MD trajectories

The Principal Component Analysis (PCA) [61] was performed with GROMACS using C^α^ atoms coordinates of snapshots extracted from the production trajectory every 100 ps. The trajectories were then projected onto the PCs associated with the two largest eigenvalues (PC1 and PC2). PCs were analysed using the DynDom [62] software, which can be used to identify dynamic domains in the protein and their relative motion. DynDom also determines hinge regions at the interface between the domains and rotational axes. A PCA was performed also on a pseudo-trajectory obtained by combining the last 100 ns of all the Apo and OM-bound trajectories.

The correlation networks were generated by calculating the dynamical cross-correlation (DCC) matrices with Wordom [63] using C^α^ atoms coordinates of snapshots extracted from the production trajectory every 100 ps. Multiple matrices were first generated by calculating time averages over 5 ns. Averaging over these matrices produced the final DCC matrix [64].

The calculation and analysis of local correlation networks was performed as described in [49]. In brief, local conformational changes and their correlation were described using the M32K25 [65, 66] Structural Alphabet (SA), namely a collection of fragments of 4 consecutive C^α^ atoms representing prototypical backbone conformations. The correlation of conformational changes in a pair of protein fragments is calculated as normalized Mutual Information (MI) between the two sequences of SA letters representing the different conformations explored by the fragments during the simulation. As previously shown, MI networks can be used to describe allosteric transmission pathways in proteins [49, 67]. In particular, transmission pathways between two regions can be identified by calculating the set of shortest paths connecting them in the MI network. If a source site is selected (e.g. an allosteric site), it is possible to detect the regions in the protein that have a preferential connection with it by identifying the fragments that are closer to the source site than the average [49]. The SA analysis was performed on C^α^ atoms coordinates extracted every 1 ps. The statistical significance of the MI values was determined by generating a random background distribution of 1000 samples as described in [49]. Fragments are labelled in the text using the first residue of the fragment.

The matrix of OM-Apo contact changes was derived by first calculating the frequency of inter-residue contacts for each Apo and OM-bound simulation. Two residues were considered to be in contact if the minimum distance calculated over all the pairs of non-hydrogen atoms was < 4 Å. A consensus contact matrix was then calculated from the four simulations of each binding state (S1 Text). The final matrix of OM-Apo contact changes was derived by subtracting the Apo consensus matrix from the OM one and calculating the absolute value, so that elements different from 0 indicate contacts that are either stabilised or destabilised upon OM binding.

In order to remove the noise from the modelled loops, most of which showed a high flexibility (S7 Fig), the PCs and DDC, MI and contact change matrices were calculated considering only the residues solved in the starting X-ray structures (see S8 Table for a list of the excluded residues). All the residues in the protein were used for the RMSD, RMSF and secondary structure analyses.

The analyses were performed using GROMACS and GSATools [68], together with in-house R scripts using the Bio3D [69] library. Networks were visualised with the xPyder [70] plugin for PyMOL. A more detailed description of the analyses performed for this work can be found in S1 Text.

## Supporting information

S1 Table Overview of the simulations. (PDF)

S2 Table Contribution to the total variance (%) for PC1, PC2 and PC1+PC2. (PDF)

S3 Table Frequency of OM-residue contacts during OM-bound MD simulations. (PDF)

S4 Table Frequency of OM-residue hydrogen bonds during OM-bound MD simulations. (PDF)

S5 Table Cluster analysis of OM conformations. (PDF)

S6 Table Node degeneracy in the OM-Apo contact change network. (PDF)

S7 Table OM-Apo contact frequency differences. (PDF)

S8 Table Regions modelled in the Apo and OM-bound systems. (PDF)

S9 Table OM GAFF atom types and AM1-BCC partial atomic charges. (PDF)

S10 Table Performance comparison for sp^2^ and sp^3^ parameter sets of N05. (PDF)

S1 Fig Collective motions in Apo simulations. Porcupine representation of the first two Principal Components in the Apo simulations. The orange spikes show the direction and relative amplitude of motion of each residue along the PC. The approximate direction of the CLD hinge axis is also shown when relevant.
(PDF)

S2 Fig OM conformational dynamics. A. Time evolution of the OM-protein distance for the OM-bound simulations. The distance was calculated as the minimum value over all possible pairs of non-hydrogen atoms. B. Distribution of OM RMSD values calculated between the MD structures and the X-ray structures (chain A for OMA1/2 and chain B for OMB1/2). The RMSD values for the representative structures of the first 4 most populated clusters are reported as coloured dots (see below for the colour scheme). C. Superimposition of OM structures represented as blue (X-ray, chain A), light blue (X-ray chain B), red (cluster 1), orange (cluster 2), yellow (cluster 3) and green (cluster 4) structures. The overall cluster population is reported in parentheses.
(PDF)

S3 Fig Hydrogen bonding interactions between OM and cMotorD. Representative snapshots from the simulations are shown to illustrate hydrogen bonding interactions between OM and residues A91, S118 and C705 (A and B), N711 (A), R712 (B), and L120 and K762 (C). The frequency of each interaction is reported in S4 Table.
(PDF)

S4 Fig Network of inter-residue contacts in the OM-binding site. Red edges connect pair of residues that are found in contact for at least 70% of the simulation. All the residues within 8 Å from OM were included in the analysis.
(PDF)

S5 Fig Comparison of CLD orientations in chain A and chain B experimental structures. A superimposition of the CLD from the OM-bound chain A (blue) and chain B (light blue) experimental structures of human cardiac myosin (PDB ID: 4PA0) is shown, together with a structure of the pre-power stroke state (magenta, PDB ID: 1BR1) from chicken smooth muscle for comparison.
(PDF)

S6 Fig Stability of chain A and chain B conformations of the Lever Arm in OM-bound simulations. The time evolution of the C^α^ RMSD of the Lever Arm helix from the initial structures is reported. For each OM-bound simulation, the RMSD is calculated from chain A (RMSDA, orange) and chain B (RMSDB, blue) initial structures.
(PDF)

S7 Fig Profiles of C^α^ RMSF. The RMSF (Å) is reported for Apo (green) and OM-bound (blue) simulations. Coloured blocks indicate the position in the sequence of N-terminal (green), U50K (red), L50K (grey) and CLD (blue) subdomains together with the relay helix (cyan). The position of the modelled loops (S8 Table) is indicated with transparent blocks.
(PDF)

S8 Fig Collective motions in OM-bound simulations. Porcupine representation of the first two Principal Components in the OM-bound simulations. The orange spikes show the direction and relative amplitude of motion of each residue along the PC.
(PDF)

S9 Fig Projection of the Apo and OM-bound trajectories onto the first two Principal Components. Each trajectory (production phase) is projected onto its corresponding PCs (S1 and S8 Figs), with points coloured according to time from blue (t = 0 ns) to red (t = 300 ns). Projections are reported in Å. The contribution of each PC to the total variance is reported in the axis label.
(PDF)

S10 Fig PCA on the combined Apo and OM-bound trajectories. The PCA was performed on a pseudo-trajectory obtained by concatenating the last 100 ns from all the Apo and OM-bound trajectories. A. Porcupine representation of the first two Principal Components. The orange spikes show the direction and relative amplitude of motion of each residue along the PC. B. Projection of Apo (green hues) and OM-bound (blue hues) trajectories onto PC1 and PC2 (Å). The contribution of each PC to the total variance is reported in the axis label.
(PDF)

S11 Fig Comparison of representative structures from Apo and OM-bound trajectories. Cartoon representation of structures sampled at the end of ApoA1 (A) and OMA2 (B) simulations (280 ns). The CLD (blue cartoon) and key residues in the OM-binding region (orange sticks) are highlighted.
(PDF)

S12 Fig Dynamical cross-correlation matrices (DCCM). DCCM values are reported for Apo (left) and OM-bound (middle) simulations, together with OM-Apo differences (ΔDCCM, right). Differences larger than 0.16 in absolute value are shown in red (positive values) and green (negative values). The ΔDCCM threshold and the colour code are the same as Fig 7. Residues in the modelled loops were not considered in the analysis and are not reported.
(PDF)

S13 Fig Preferential connections in the local correlation network between the OM-binding site and functional regions in cMotorD. Plots of the difference between OM and Apo preferential connection scores Δζ calculated using different residues from the OM-binding site as source sites. The source site is indicated on top of each profile. A negative value indicates a stronger preferential connection to the source residue in OM-bound simulations compared to Apo ones. Negative bars are coloured according to the subdomain and grey areas highlight the position of functional regions (see Fig 8 caption for a legend). Residues were selected by clustering the profiles generated for all the residues in the OM-binding site. Cluster representatives are shown here to illustrate the range of variability of the profiles.
(PDF)

S14 Fig Profiles of preferential connection scores ζ calculated using V698 as source site. Scores calculated from Apo (top) and OM-bound (bottom) consensus MI matrices are reported using the colour code described in Fig 8 caption. Grey areas highlight the position of functional regions (see Fig 8 caption for a legend).
(PDF)

S15 Fig Structural formula of omecamtiv mecarbil (OM) with atom numbering. (PDF)

S16 Fig Time evolution of C^α^ RMSD. The RMSD (Å) values from the initial structure are reported for each Apo (green hues, top panels) and OM-bound (blue hues, bottom panel) simulation.
(PDF)

S17 Fig Time evolution of the secondary structure. The DSSP annotation is reported for each simulation, with coloured blocks indicating α-helices (blue), β-strands (red), β-bridges (black), bends (green), turns (yellow), π-helices (purple) and 3_10_-helices (grey).
(PDF)

S1 Text Supplementary methods. (PDF)
