# Supplementary material for: Allosteric modulation of cardiac myosin dynamics by omecamtiv mecarbil
Source: PLoS Comput Biol. 2017 Nov 6;13(11):e1005826. doi: 10.1371/journal.pcbi.1005826 (PMC5690683; doi:10.1371/journal.pcbi.1005826)
Supplement: S1 Table — (PDF) [file pcbi.1005826.s001.pdf]

S1 Table. Overview of the simulations.

Starting structure[a]	Label[b]	Simulation length (ns)
Apo, chain A, loop model 1	ApoA1	300
Apo, chain A, loop model 2	ApoA2	300
Apo, chain B, loop model 1	ApoB1	300
Apo, chain B, loop model 2	ApoB2	300
OM-bound, chain A, loop model 1	OMA1	300
OM-bound, chain A, loop model 2	OMA2	300
OM-bound, chain B, loop model 1	OMB1	300
OM-bound, chain B, loop model 2	OMB2	300

[a]Four replicas were run for each binding state (Apo and OM-bound), starting from two different chains in the X-ray structure (chain A and B) combined with two alternative models for the loops with missing experimental coordinates (1 and 2).

[b]Each simulation is labelled in the main text by adding the chain (A or B) and the loop model number (1 or 2) to the binding state label (Apo or OM).
