# Supplementary material for: Allosteric modulation of cardiac myosin dynamics by omecamtiv mecarbil
Source: PLoS Comput Biol. 2017 Nov 6;13(11):e1005826. doi: 10.1371/journal.pcbi.1005826 (PMC5690683; doi:10.1371/journal.pcbi.1005826)
Supplement: S2 Table — (PDF) [file pcbi.1005826.s002.pdf]

S2 Table. Contribution to the total variance (%) for PC1, PC2 and PC1+PC2

	Apo		
	**PC1**	**PC2**	**PC1+PC2**
A1	43	13	56
A2	31	14	45
B1	56	9	65
B2	23	11	33
	**OM**		
	**PC1**	**PC2**	**PC1+PC2**
A1	16	9	25
A2	22	10	32
B1	30	9	38
B2	16	9	26
