# Supplementary material for: Allosteric modulation of cardiac myosin dynamics by omecamtiv mecarbil
Source: PLoS Comput Biol. 2017 Nov 6;13(11):e1005826. doi: 10.1371/journal.pcbi.1005826 (PMC5690683; doi:10.1371/journal.pcbi.1005826)
Supplement: S3 Table — (PDF) [file pcbi.1005826.s003.pdf]

S3 Table. Frequency of OM-residue contacts during OM-bound MD simulations

Residue[a]	OMA1[b]	OMA2[b]	OMB1[b]	OMB2[b]
F84	0.17 (0.17)	0.08 (0.08)	0.25 (0.25)	0.60 (0.60)
M90	0.50 (0.50)	0.97 (0.68)	0.55 (0.50)	0.99 (0.92)
A91	0.96 (0.84)	1.00 (0.97)	1.00 (0.96)	1.00 (0.93)
M92	0.83 (0.60)	1.00 (0.93)	0.98 (0.85)	1.00 (0.94)
L93	0.16 (0.00)	0.52 (0.00)	0.60 (0.00)	0.81 (0.01)
T94	0.15 (0.11)	0.56 (0.51)	0.60 (0.43)	0.75 (0.67)
L96	0.70 (0.70)	0.86 (0.86)	0.80 (0.80)	0.83 (0.83)
V101	0.00 (0.00)	0.16 (0.16)	0.08 (0.08)	0.39 (0.39)
S118	0.99 (0.57)	0.97 (0.85)	0.88 (0.72)	0.96 (0.85)
G119	0.89 (0.00)	0.60 (0.00)	0.35 (0.00)	0.33 (0.00)
L120	0.39 (0.30)	0.04 (0.03)	0.07 (0.07)	0.05 (0.04)
F121	0.28 (0.28)	0.00 (0.00)	0.81 (0.81)	0.32 (0.32)
F489	0.13 (0.13)	0.00 (0.00)	0.00 (0.00)	0.00 (0.00)
M493	0.54 (0.54)	0.62 (0.62)	0.59 (0.59)	0.60 (0.60)
E497	0.13 (0.13)	0.30 (0.30)	0.67 (0.67)	0.50 (0.50)
E500	0.08 (0.08)	0.01 (0.01)	0.21 (0.21)	0.04 (0.04)
V698	0.90 (0.26)	0.01 (0.00)	0.51 (0.24)	0.05 (0.01)
G701	0.52 (0.00)	0.61 (0.00)	0.97 (0.00)	0.47 (0.00)
I702	0.71 (0.63)	0.91 (0.91)	0.96 (0.82)	0.97 (0.97)
C705	0.94 (0.94)	0.96 (0.96)	0.87 (0.77)	1.00 (1.00)
P710	0.90 (0.10)	1.00 (0.15)	1.00 (0.36)	1.00 (0.15)
N711	0.28 (0.22)	0.96 (0.85)	0.99 (0.48)	0.95 (0.50)
R712	0.94 (0.93)	1.00 (0.99)	1.00 (1.00)	1.00 (1.00)
I713	0.10 (0.09)	0.77 (0.77)	0.09 (0.09)	0.13 (0.13)
K762	0.36 (0.36)	0.03 (0.03)	0.02 (0.02)	0.03 (0.03)
L770	0.15 (0.15)	0.59 (0.59)	0.47 (0.47)	0.48 (0.48)

[a]Only residues with frequency >= 0.1 are reported.

[b]A residue is considered in contact with OM if the OM-residue minimum distance calculated over non-hydrogen atoms is < 4 Å. Values reported in parentheses are calculated considering only the atoms in the side chains.
