# Supplementary material for: Allosteric modulation of cardiac myosin dynamics by omecamtiv mecarbil
Source: PLoS Comput Biol. 2017 Nov 6;13(11):e1005826. doi: 10.1371/journal.pcbi.1005826 (PMC5690683; doi:10.1371/journal.pcbi.1005826)
Supplement: S4 Table — (PDF) [file pcbi.1005826.s004.pdf]

S4 Table. Frequency of OM-residue hydrogen bonds during OM-bound MD simulations

**Residue**	**OMA1[a]**	**OMA2[a]**	**OMB1[a]**	**OMB2[a]**
R712	0.18	0.47	0.61	0.31
C705	0.04	0.50	0.02	0.47
A91	0.11	0.22	0.25	0.15
N711	0.09	0.22	0.22	0.00
S118	0.06	0.12	0.00	0.10
K762	0.21	0.00	0.01	0.01
L120	0.19	0.00	0.00	0.00

[a]Hydrogen bonds are calculated using a threshold of 3.5 Å on the D(onor)-A(cceptor) distance and 30° on the D-H-A angle.
