# Supplementary material for: Allosteric modulation of cardiac myosin dynamics by omecamtiv mecarbil
Source: PLoS Comput Biol. 2017 Nov 6;13(11):e1005826. doi: 10.1371/journal.pcbi.1005826 (PMC5690683; doi:10.1371/journal.pcbi.1005826)
Supplement: S5 Table — (PDF) [file pcbi.1005826.s005.pdf]

S5 Table. Cluster analysis of OM conformations.

# cluster	population[a] (%)	$RMSD_A$[b] (Å)	$RMSD_B$[b] (Å)	OMA1[c] (%)	OMA2[c] (%)	OMB1[c] (%)	OMB2[c] (%)	representative frame[d]
1	82.4	2.20	2.11	12.0	24.7	20.8	24.8	265 ns (OMA2)
2	8.3	2.88	2.60	8.3	0.0	0.0	0.0	127 ns (OMA1)
3	4.5	2.27	2.05	0.3	0.2	4.1	0.0	292 ns (OMB1)
4	1.8	7.39	6.95	1.8	0.0	0.0	0.0	297 ns (OMA1)

[a]Fraction of the concatenated OM-bound trajectory belonging to each cluster.

[b]RMSD from the OM X-ray structure in chain A ($RMSD_A$) and chain B ($RMSD_B$) calculated over OM non-hydrogen atoms. The fitting of the structures was performed using all the C^{α} atoms in the protein.

[c]Contribution to the cluster population from each OM-bound simulation.

[d]Representative frame for each cluster (the corresponding trajectory is reported in parentheses).
