# Supplementary material for: Allosteric modulation of cardiac myosin dynamics by omecamtiv mecarbil
Source: PLoS Comput Biol. 2017 Nov 6;13(11):e1005826. doi: 10.1371/journal.pcbi.1005826 (PMC5690683; doi:10.1371/journal.pcbi.1005826)
Supplement: S6 Table — (PDF) [file pcbi.1005826.s006.pdf]

S6 Table. Node degeneracy in the OM-Apo contact change network

Resid[a]	Degeneracy[b]
121	0.40
174	0.20
175	0.17
176	0.20
177	0.52
178	0.31
184	0.23
191	0.20
195	0.20
217	0.15
220	0.11
223	0.57
226	0.23
227	0.65
246	0.72
258	0.15
266	0.72
338	0.15
459	0.20
462	0.72
671	0.17
693	0.43
696	0.83
698	1.00

[a]Residues involved in the shortest paths connecting V698 and G helix residues in the network of OM-Apo contact changes (Fig. 10).

[b]Fraction of the number of paths going through each residue.
