# Supplementary material for: Allosteric modulation of cardiac myosin dynamics by omecamtiv mecarbil
Source: PLoS Comput Biol. 2017 Nov 6;13(11):e1005826. doi: 10.1371/journal.pcbi.1005826 (PMC5690683; doi:10.1371/journal.pcbi.1005826)
Supplement: S7 Table — (PDF) [file pcbi.1005826.s007.pdf]

S7 Table. OM-Apo contact frequency difference

i[a]	j[a]	Δf[b]
195	199	0.95
178	696	-0.68
199	258	0.58
177	696	-0.56
195	258	-0.52
199	252	0.45
464	475	-0.40
217	258	0.36
466	475	-0.35
195	252	-0.34
174	195	0.28
222	226	-0.27
191	459	-0.24
180	466	-0.24
223	227	-0.22
176	459	0.21
177	462	0.21
224	246	-0.21
177	464	0.21
175	671	0.21
693	696	0.20
216	261	0.20
185	242	-0.20
223	338	0.19
176	184	-0.17
195	250	-0.17
220	223	-0.17
174	191	-0.16
217	221	-0.16
246	266	0.15
218	338	-0.15
228	242	0.15
266	462	-0.15
178	462	0.15
178	464	0.15
121	698	0.15
216	259	-0.14
180	242	-0.14
121	696	0.14
339	439	0.14
223	226	0.14
230	439	-0.13
219	338	-0.12
226	339	-0.12
250	261	-0.12
185	461	-0.12
693	698	-0.12
184	461	0.11
175	462	-0.11
671	698	-0.11
252	259	0.11
177	184	-0.11
227	246	-0.11
220	258	-0.10

[a]Residues involved in the shortest paths connecting V698 and G helix residues in the network of OM-Apo contact changes (Fig. 10).

[b]Difference between the consensus matrices of contact frequencies calculated from the OM-bound and Apo simulations.
