# Supplementary material for: Allosteric modulation of cardiac myosin dynamics by omecamtiv mecarbil
Source: PLoS Comput Biol. 2017 Nov 6;13(11):e1005826. doi: 10.1371/journal.pcbi.1005826 (PMC5690683; doi:10.1371/journal.pcbi.1005826)
Supplement: S8 Table — (PDF) [file pcbi.1005826.s008.pdf]

S8 Table. Regions modelled in the Apo and OM-bound systems

System	Actin-binding region		Converter
	Lower 50 kDa domain	Upper 50 kDa domain	
ApoA	204-210, 369-370, 404-410	625-644	732-735
ApoB	204-210, 369-370, 404-410	625-644	717-737
OMA	206-208, 406-411	625-643	-
OMB	205-209, 405-411	625-643	730-737, 749-753
Modelled regions (union)[a]	204-210, 404-411	625-644	723-737

[a] Definition of regions that were excluded from selected analyses (Methods).
