# Supplementary material for: Allosteric modulation of cardiac myosin dynamics by omecamtiv mecarbil
Source: PLoS Comput Biol. 2017 Nov 6;13(11):e1005826. doi: 10.1371/journal.pcbi.1005826 (PMC5690683; doi:10.1371/journal.pcbi.1005826)
Supplement: S9 Table — (PDF) [file pcbi.1005826.s009.pdf]

S9 Table. OM GAFF atom types and AM1-BCC partial atomic charges.

Atom name[a]	Atom type	Atomic Charges[b]	
		Chain A	Chain B
C01	c3	0.137	0.136
H01	h1	0.052	0.052
H02	h1	0.052	0.052
H03	h1	0.052	0.052
O02	os	-0.441	-0.437
C03	c	0.721	0.726
O04	o	-0.580	-0.587
N05	n	-0.403	-0.417
C06	c3	0.071	0.077
H04	h1	0.069	0.067
H05	h1	0.069	0.067
C07	c3	0.146	0.152
H06	h1	0.047	0.048
H07	h1	0.047	0.048
N08	n3	-0.733	-0.737
C09	c3	0.219	0.222
H08	h1	0.068	0.066
H09	h1	0.068	0.066
C10	ca	-0.130	-0.130
C11	ca	-0.125	-0.125
H10	ha	0.140	0.140
C12	ca	-0.118	-0.119
H11	ha	0.143	0.143
C13	ca	-0.119	-0.119
H12	ha	0.178	0.178
C14	ca	0.042	0.042
N15	n	-0.462	-0.460
H13	hn	0.332	0.331
C16	c	0.778	0.778
O17	o	-0.621	-0.620
N18	n	-0.474	-0.474
H14	hn	0.342	0.342
C19	ca	-0.086	-0.086
C20	ca	0.381	0.381
H15	h4	0.034	0.037
N21	nb	-0.650	-0.650
C22	ca	0.405	0.404
C23	c3	-0.149	-0.149
H16	hc	0.056	0.056
H17	hc	0.056	0.056
H18	hc	0.056	0.056
C24	ca	-0.227	-0.227
H19	ha	0.148	0.148
C25	ca	-0.121	-0.121
H20	ha	0.144	0.144
C26	ca	0.075	0.075
F27	f	-0.147	-0.148
C28	c3	0.146	0.152
H21	h1	0.047	0.048
H22	h1	0.047	0.048
C29	c3	0.071	0.077
H23	h1	0.069	0.067
H24	h1	0.069	0.067

[a] See S15 Fig for atom numbering.
[b] Charges are reported in a.u.
