# Supplementary material for: Allosteric modulation of cardiac myosin dynamics by omecamtiv mecarbil
Source: PLoS Comput Biol. 2017 Nov 6;13(11):e1005826. doi: 10.1371/journal.pcbi.1005826 (PMC5690683; doi:10.1371/journal.pcbi.1005826)
Supplement: S10 Table — (PDF) [file pcbi.1005826.s010.pdf]

S10 Table. Performance comparison for sp^2 and sp^3 parameter sets of N05

	Chain A		Chain B	
	sp^2	sp^3	sp^2	sp^3
$RMSD_{ring}$ (Å)[a]	0.24	0.34	0.22	0.18
$RMSD_{dihe}$ (°)[b]	21.3	70.5	22.1	87.1

[a] RMSD values between the X-ray and energy minimised structures calculated over the non-hydrogen atoms of the piperazine ring + C03 and C09 (S15 Fig)

[b] RMSD of N05 dihedral angles from the reference X-ray values calculated over short test simulations. RMSD values were first calculated singularly for two selected dihedrals involving N05 (O04-C03-N05-C06 and C03-C06-N05-C29) and then averaged.
