# Supplementary material for: Allosteric modulation of cardiac myosin dynamics by omecamtiv mecarbil
Source: PLoS Comput Biol. 2017 Nov 6;13(11):e1005826. doi: 10.1371/journal.pcbi.1005826 (PMC5690683; doi:10.1371/journal.pcbi.1005826)
Supplement: S1 Fig — Porcupine representation of the first two Principal Components in the Apo simulations. The orange spikes show the direction and relative amplitude of motion of each residue along the PC. The approximate direction of the CLD hinge axis is also shown when relevant. (PDF) [file pcbi.1005826.s011.pdf]

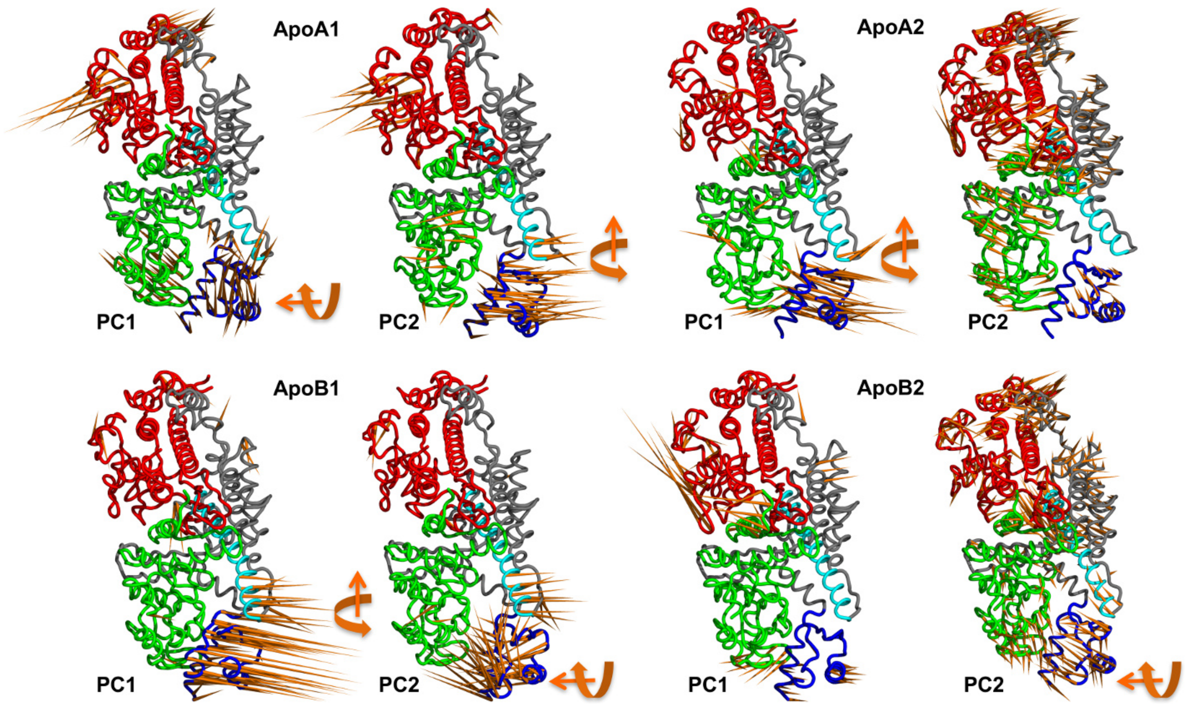

S1 Fig. Collective motions in Apo simulations. Porcupine representation of the first two Principal Components in the Apo simulations. The orange spikes show the direction and relative amplitude of motion of each residue along the PC. The approximate direction of the CLD hinge axis is also shown when relevant.
