# Supplementary material for: Allosteric modulation of cardiac myosin dynamics by omecamtiv mecarbil
Source: PLoS Comput Biol. 2017 Nov 6;13(11):e1005826. doi: 10.1371/journal.pcbi.1005826 (PMC5690683; doi:10.1371/journal.pcbi.1005826)
Supplement: S2 Fig — A. Time evolution of the OM-protein distance for the OM-bound simulations. The distance was calculated as the minimum value over all possible pairs of non-hydrogen atoms. B. Distribution of OM RMSD values calculated between the MD structures and the X-ray structures (chain A for OMA1/2 and chain B for OMB1/2). The RMSD values for the representative structures of the first 4 most populated clusters are reported as coloured dots (see below for the colour scheme). C. Superimposition of OM structures represented as blue (X-ray, chain A), light blue (X-ray chain B), red (cluster 1), orange (cluster 2), yellow (cluster 3) and green (cluster 4) structures. The overall cluster population is reported in parentheses. (PDF) [file pcbi.1005826.s012.pdf]

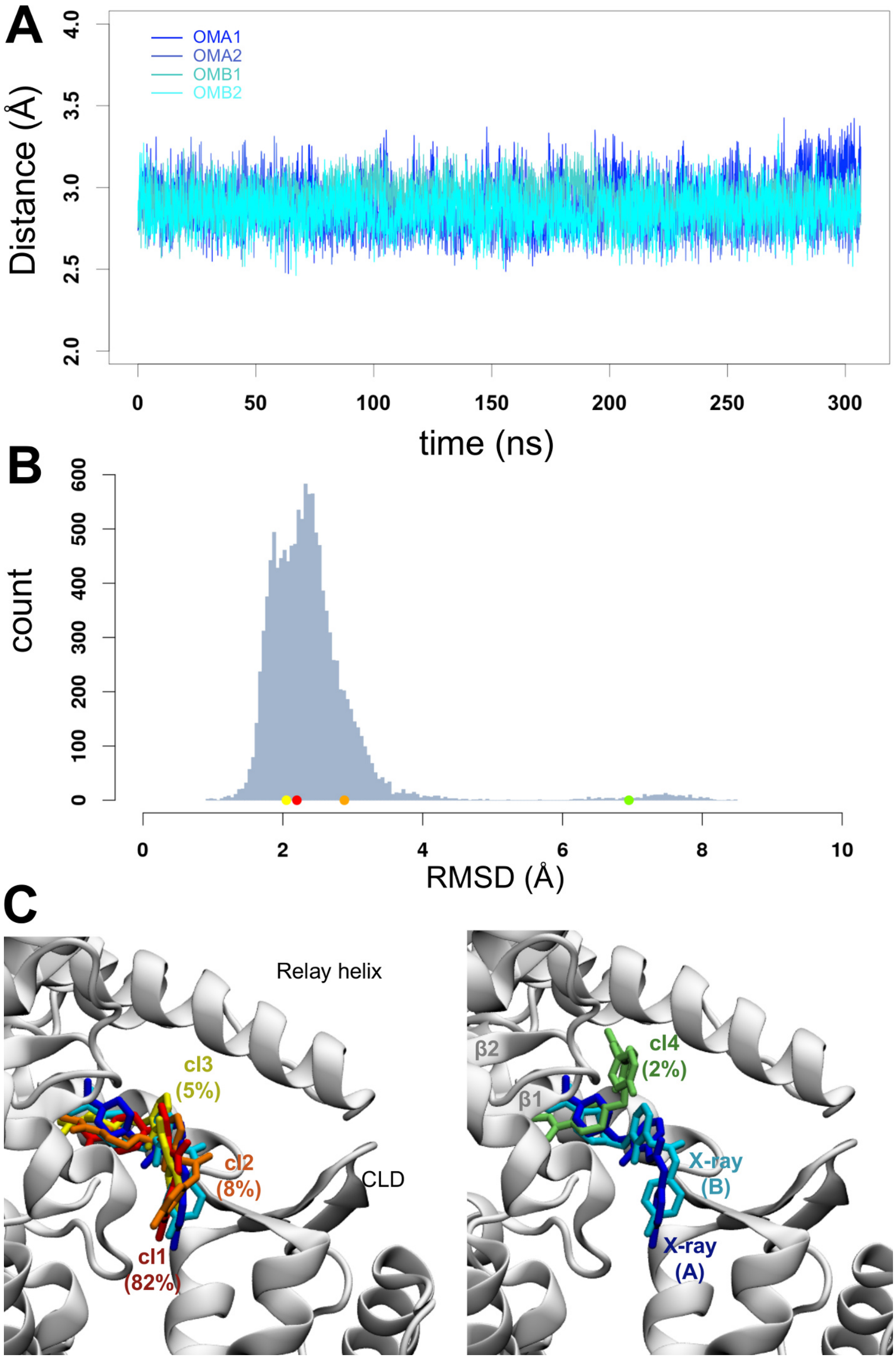

S2 Fig. OM conformational dynamics. A. Time evolution of the OM-protein distance for the OM-bound simulations. The distance was calculated as the minimum value over all possible pairs of non-hydrogen atoms. B. Distribution of OM RMSD values calculated between the MD structures and the X-ray structures (chain A for OMA1/2 and chain B for OMB1/2). The RMSD values for the representative structures of the first 4 most populated clusters are reported as coloured dots (see below for the colour scheme). C. Superimposition of OM structures represented as blue (X-ray, chain A), light blue (X-ray chain B), red (cluster 1), orange (cluster 2), yellow (cluster 3) and green (cluster 4) structures. The overall cluster population is reported in parentheses.
