# Supplementary material for: Allosteric modulation of cardiac myosin dynamics by omecamtiv mecarbil
Source: PLoS Comput Biol. 2017 Nov 6;13(11):e1005826. doi: 10.1371/journal.pcbi.1005826 (PMC5690683; doi:10.1371/journal.pcbi.1005826)
Supplement: S3 Fig — Representative snapshots from the simulations are shown to illustrate hydrogen bonding interactions between OM and residues A91, S118 and C705 (A and B), N711 (A), R712 (B), and L120 and K762 (C). The frequency of each interaction is reported in S4 Table. (PDF) [file pcbi.1005826.s013.pdf]

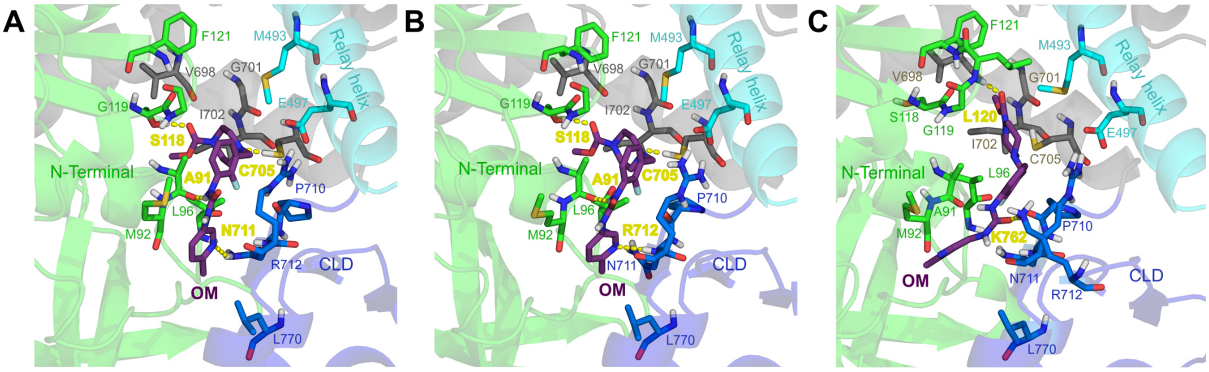

S3 Fig. Hydrogen bonding interactions between OM and cMotorD. Representative snapshots from the simulations are shown to illustrate hydrogen bonding interactions between OM and residues A91, S118 and C705 (A and B), N711 (A), R712 (B), and L120 and K762 (C). The frequency of each interaction is reported in S4 Table.
