# Supplementary material for: Allosteric modulation of cardiac myosin dynamics by omecamtiv mecarbil
Source: PLoS Comput Biol. 2017 Nov 6;13(11):e1005826. doi: 10.1371/journal.pcbi.1005826 (PMC5690683; doi:10.1371/journal.pcbi.1005826)
Supplement: S4 Fig — Red edges connect pair of residues that are found in contact for at least 70% of the simulation. All the residues within 8 Å from OM were included in the analysis. (PDF) [file pcbi.1005826.s014.pdf]

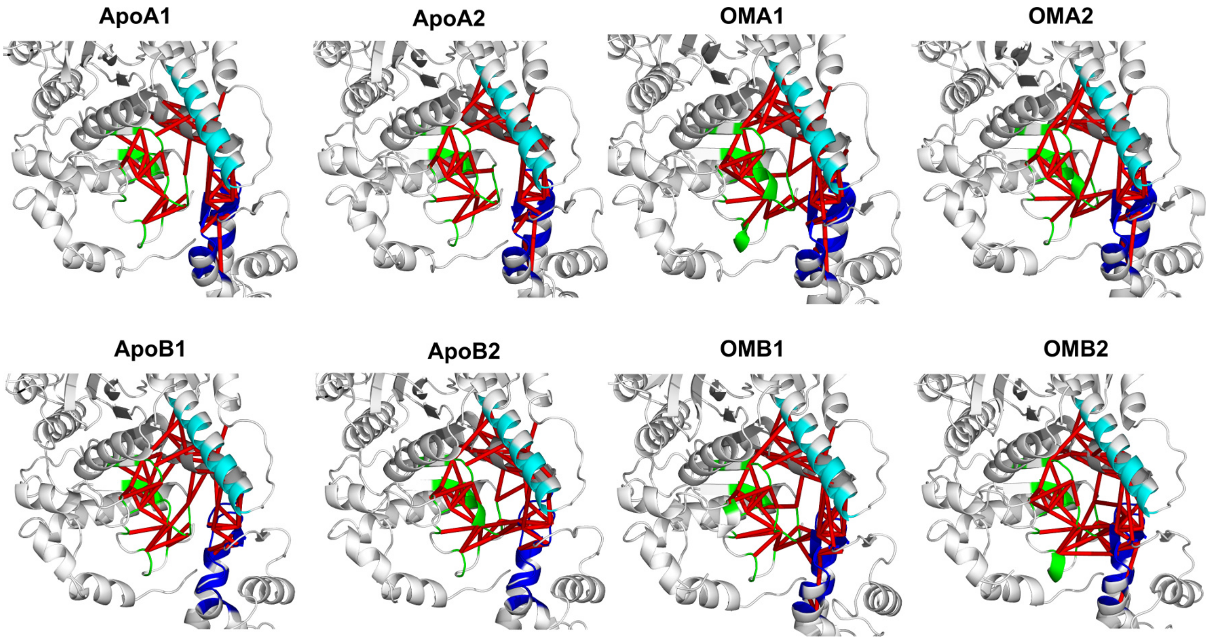

S4 Fig. Network of inter-residue contacts in the OM-binding site. Red edges connect pair of residues that are found in contact for at least 70% of the simulation. All the residues within 8 Å from OM were included in the analysis.
