# Supplementary material for: Allosteric modulation of cardiac myosin dynamics by omecamtiv mecarbil
Source: PLoS Comput Biol. 2017 Nov 6;13(11):e1005826. doi: 10.1371/journal.pcbi.1005826 (PMC5690683; doi:10.1371/journal.pcbi.1005826)
Supplement: S5 Fig — A superimposition of the CLD from the OM-bound chain A (blue) and chain B (light blue) experimental structures of human cardiac myosin (PDB ID: 4PA0) is shown, together with a structure of the pre-power stroke state (magenta, PDB ID: 1BR1) from chicken smooth muscle for comparison. (PDF) [file pcbi.1005826.s015.pdf]

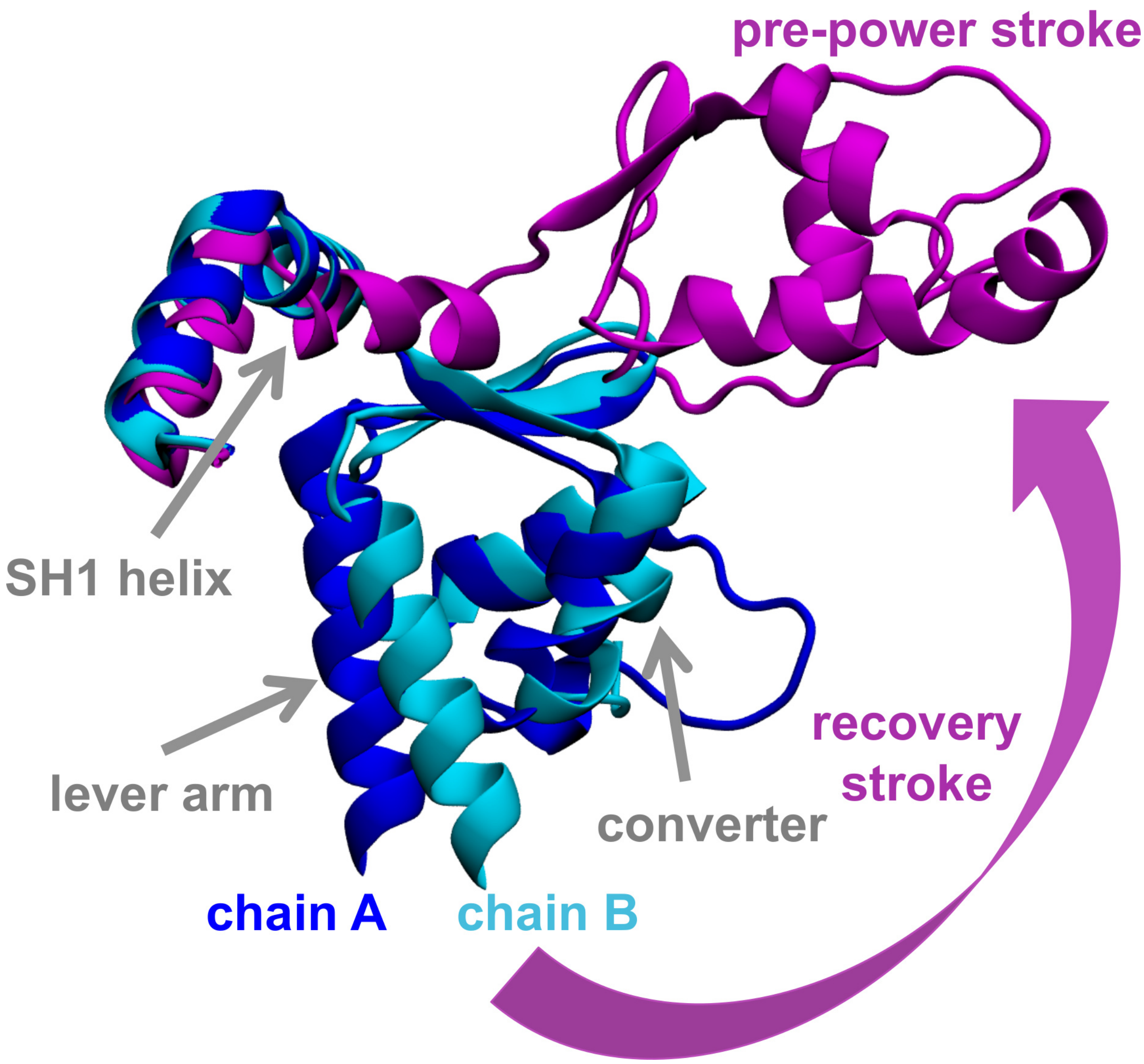


**S5 Fig. Comparison of CLD orientations in chain A and chain B experimental structures.** A superimposition of the CLD from the OM-bound chain A (blue) and chain B (light blue) experimental structures of human cardiac myosin in the near rigor conformation (PDB ID: 4PA0) is shown, together with a structure of the pre-power stroke state (magenta, PDB ID: 1BR1) from chicken smooth muscle for comparison.
