# Supplementary material for: Allosteric modulation of cardiac myosin dynamics by omecamtiv mecarbil
Source: PLoS Comput Biol. 2017 Nov 6;13(11):e1005826. doi: 10.1371/journal.pcbi.1005826 (PMC5690683; doi:10.1371/journal.pcbi.1005826)
Supplement: S6 Fig — The time evolution of the Cα RMSD of the Lever Arm helix from the initial structures is reported. For each OM-bound simulation, the RMSD is calculated from chain A (RMSDA, orange) and chain B (RMSDB, blue) initial structures. (PDF) [file pcbi.1005826.s016.pdf]

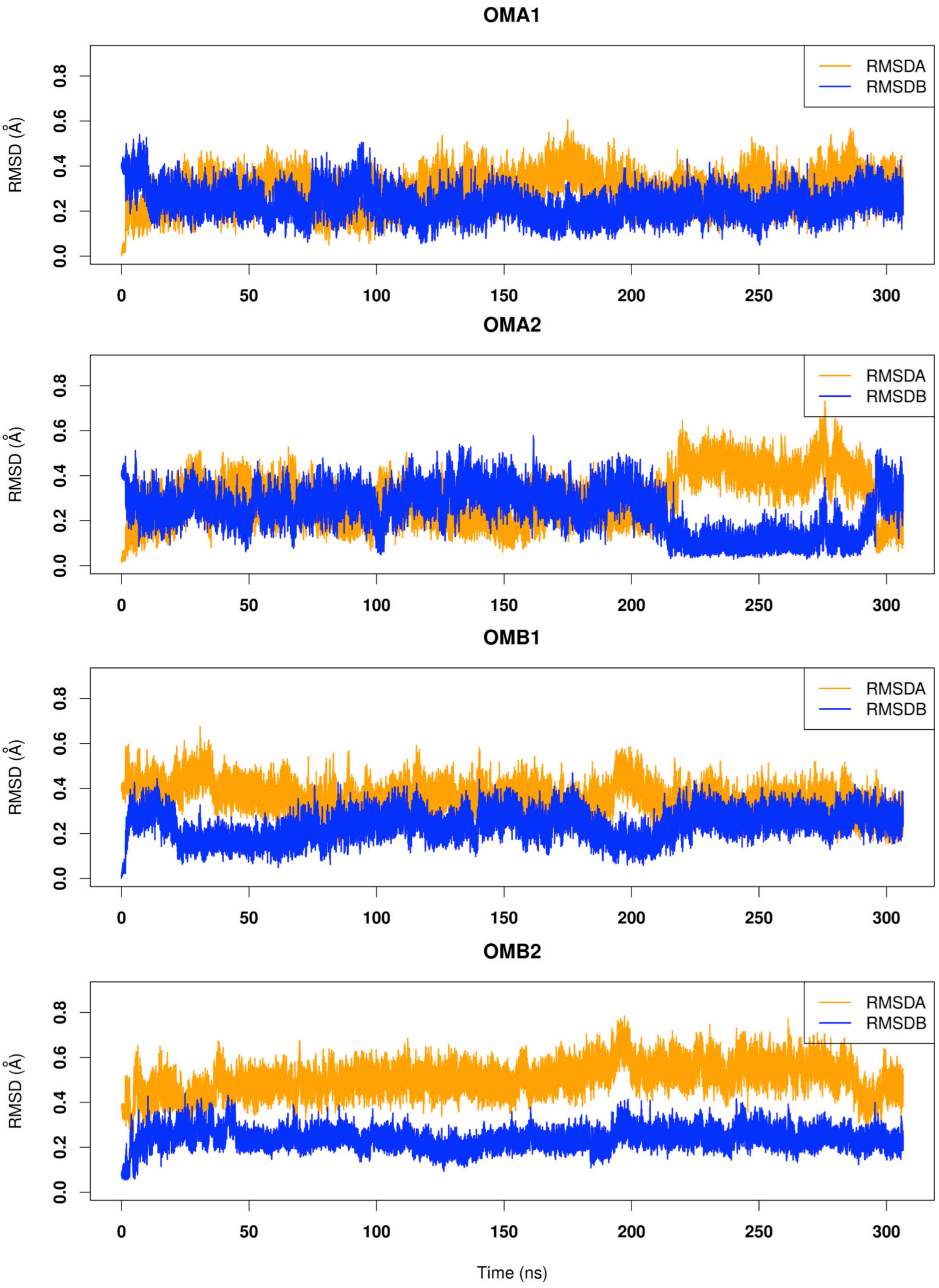

S6 Fig. Stability of chain A and chain B conformations of the Lever Arm in OM-bound simulations. The time evolution of the C^{α} RMSD of the Lever Arm helix from the initial structures is reported. For each OM-bound simulation, the RMSD is calculated from chain A (RMSDA, orange) and chain B (RMSDB, blue) initial structures.
