# Supplementary material for: Allosteric modulation of cardiac myosin dynamics by omecamtiv mecarbil
Source: PLoS Comput Biol. 2017 Nov 6;13(11):e1005826. doi: 10.1371/journal.pcbi.1005826 (PMC5690683; doi:10.1371/journal.pcbi.1005826)
Supplement: S7 Fig — The RMSF (Å) is reported for Apo (green) and OM-bound (blue) simulations. Coloured blocks indicate the position in the sequence of N-terminal (green), U50K (red), L50K (grey) and CLD (blue) subdomains together with the relay helix (cyan). The position of the modelled loops (S8 Table) is indicated with transparent blocks. (PDF) [file pcbi.1005826.s017.pdf]

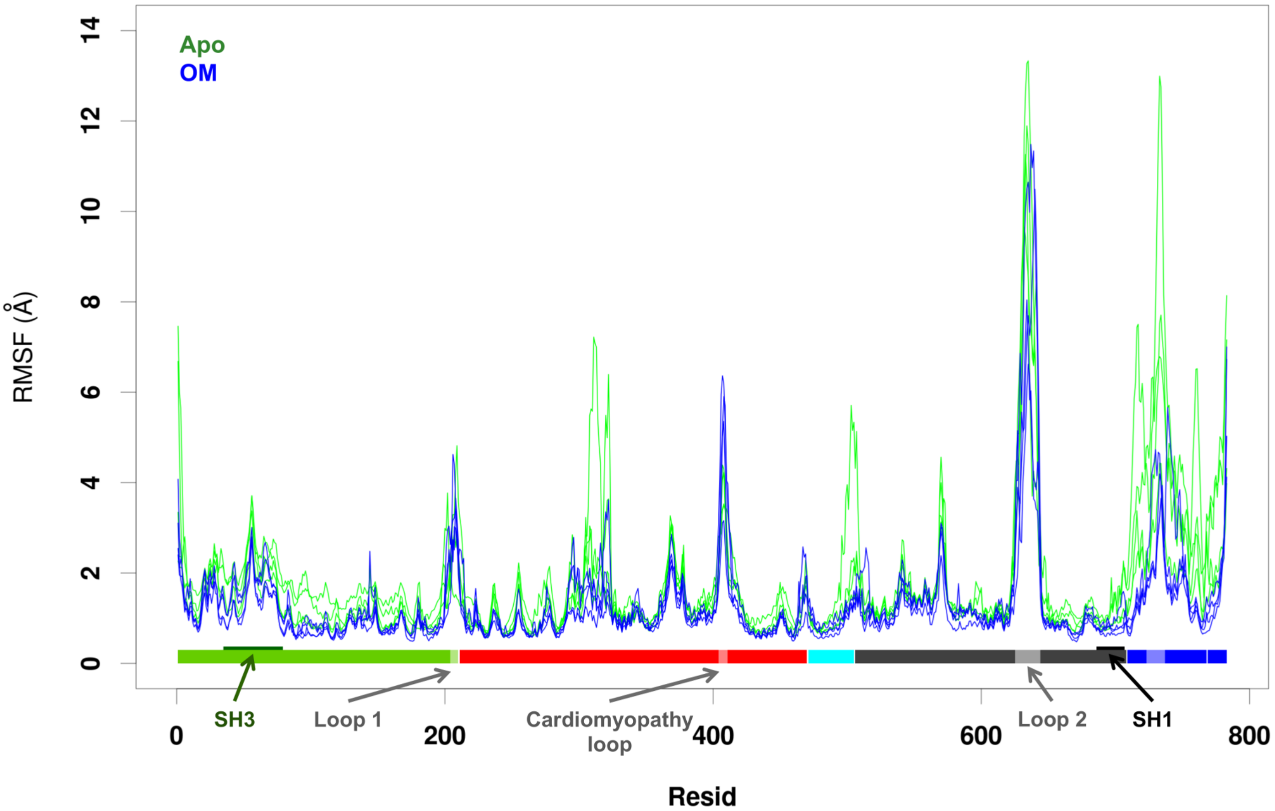

S7 Fig. Profiles of C$^{\alpha}$ RMSF. The RMSF (Å) is reported for Apo (green) and OM-bound (blue) simulations. Coloured blocks indicate the position in the sequence of N-terminal (green), U50K (red), L50K (grey) and CLD (blue) subdomains together with the relay helix (cyan). The position of the modelled loops (S8 Table) is indicated with transparent blocks, while horizontal bars indicate the position of the SH3-like domain (green) and the SH1/2 helix (black).
