# Supplementary material for: Allosteric modulation of cardiac myosin dynamics by omecamtiv mecarbil
Source: PLoS Comput Biol. 2017 Nov 6;13(11):e1005826. doi: 10.1371/journal.pcbi.1005826 (PMC5690683; doi:10.1371/journal.pcbi.1005826)
Supplement: S8 Fig — Porcupine representation of the first two Principal Components in the OM-bound simulations. The orange spikes show the direction and relative amplitude of motion of each residue along the PC. (PDF) [file pcbi.1005826.s018.pdf]

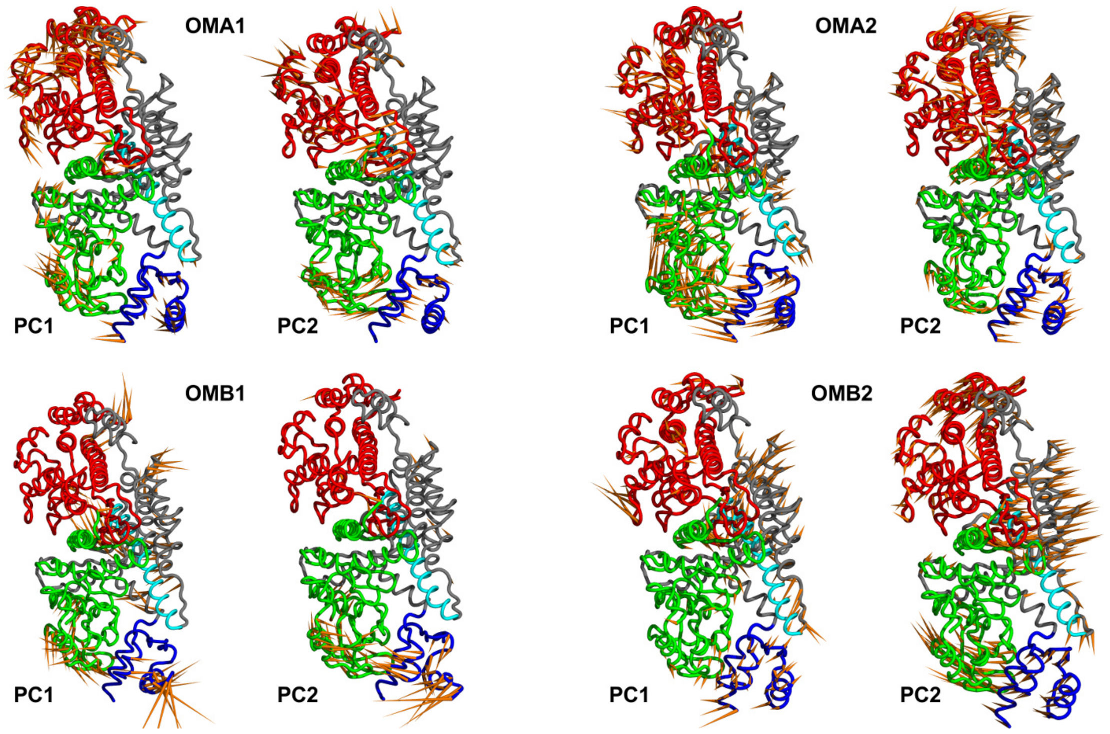

S8 Fig. Collective motions in OM-bound simulations. Porcupine representation of the first two Principal Components in the OM-bound simulations. The orange spikes show the direction and relative amplitude of motion of each residue along the PC.
