# Supplementary material for: Allosteric modulation of cardiac myosin dynamics by omecamtiv mecarbil
Source: PLoS Comput Biol. 2017 Nov 6;13(11):e1005826. doi: 10.1371/journal.pcbi.1005826 (PMC5690683; doi:10.1371/journal.pcbi.1005826)
Supplement: S9 Fig — Each trajectory (production phase) is projected onto its corresponding PCs (S1 and S8 Figs), with points coloured according to time from blue (t = 0 ns) to red (t = 300 ns). Projections are reported in Å. The contribution of each PC to the total variance is reported in the axis label. (PDF) [file pcbi.1005826.s019.pdf]

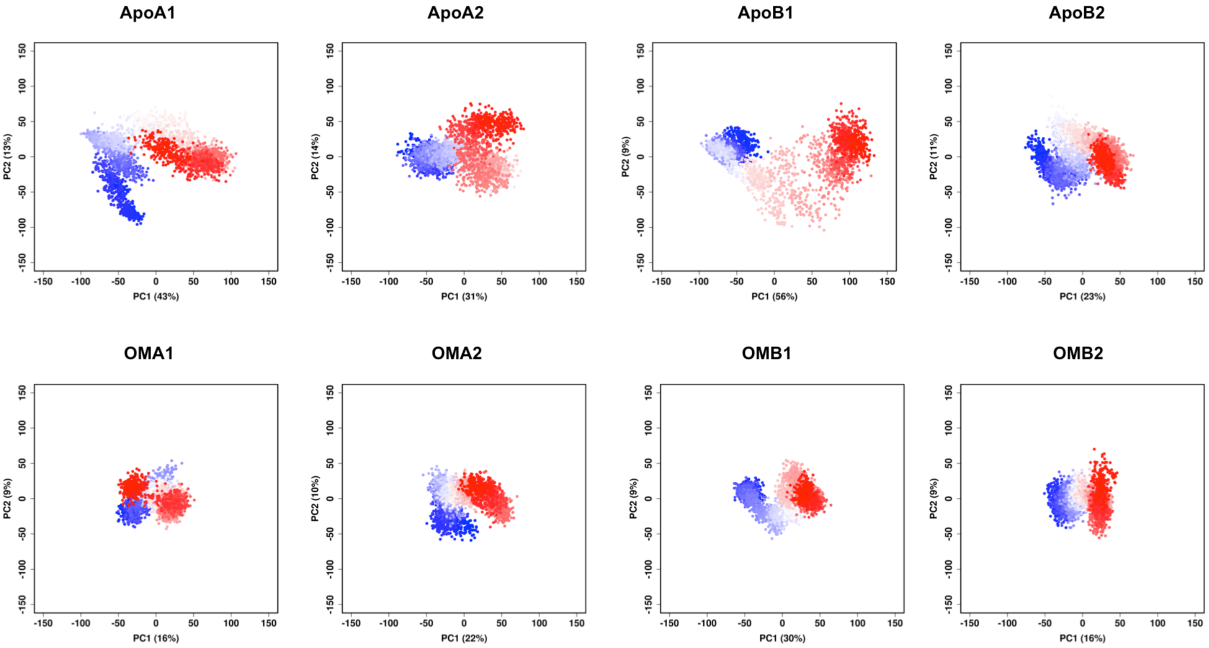

S9 Fig. Projection of the Apo and OM-bound trajectories onto the first two Principal Components. Each trajectory (production phase) is projected onto its corresponding PCs (S1 and S8 Figs), with points coloured according to time from blue (t=0 ns) to red (t = 300 ns). Projections are reported in Å. The contribution of each PC to the total variance is reported in the axis label.
