# Supplementary material for: Allosteric modulation of cardiac myosin dynamics by omecamtiv mecarbil
Source: PLoS Comput Biol. 2017 Nov 6;13(11):e1005826. doi: 10.1371/journal.pcbi.1005826 (PMC5690683; doi:10.1371/journal.pcbi.1005826)
Supplement: S10 Fig — The PCA was performed on a pseudo-trajectory obtained by concatenating the last 100 ns from all the Apo and OM-bound trajectories. A. Porcupine representation of the first two Principal Components. The orange spikes show the direction and relative amplitude of motion of each residue along the PC. B. Projection of Apo (green hues) and OM-bound (blue hues) trajectories onto PC1 and PC2 (Å). The contribution of each PC to the total variance is reported in the axis label. (PDF) [file pcbi.1005826.s020.pdf]

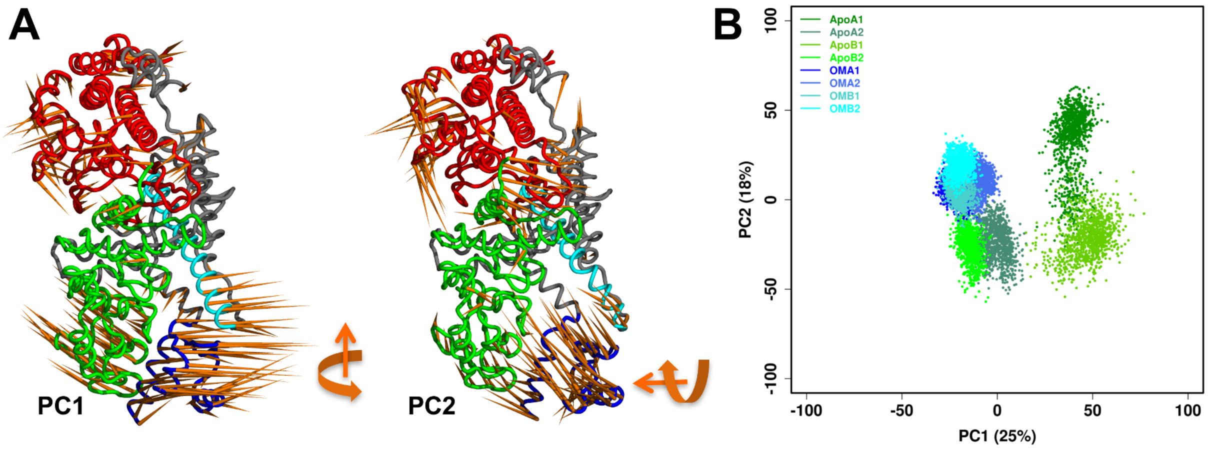

S10 Fig. PCA on the combined Apo and OM-bound trajectories. The PCA was performed on a pseudo-trajectory obtained by concatenating the last 100 ns from all the Apo and OM-bound trajectories. A. Porcupine representation of the first two Principal Components. The orange spikes show the direction and relative amplitude of motion of each residue along the PC. B. Projection of Apo (green hues) and OM-bound (blue hues) trajectories onto PC1 and PC2 (Å). The contribution of each PC to the total variance is reported in the axis label.
