# Supplementary material for: Allosteric modulation of cardiac myosin dynamics by omecamtiv mecarbil
Source: PLoS Comput Biol. 2017 Nov 6;13(11):e1005826. doi: 10.1371/journal.pcbi.1005826 (PMC5690683; doi:10.1371/journal.pcbi.1005826)
Supplement: S11 Fig — Cartoon representation of structures sampled at the end of ApoA1 (A) and OMA2 (B) simulations (280 ns). The CLD (blue cartoon) and key residues in the OM-binding region (orange sticks) are highlighted. (PDF) [file pcbi.1005826.s021.pdf]

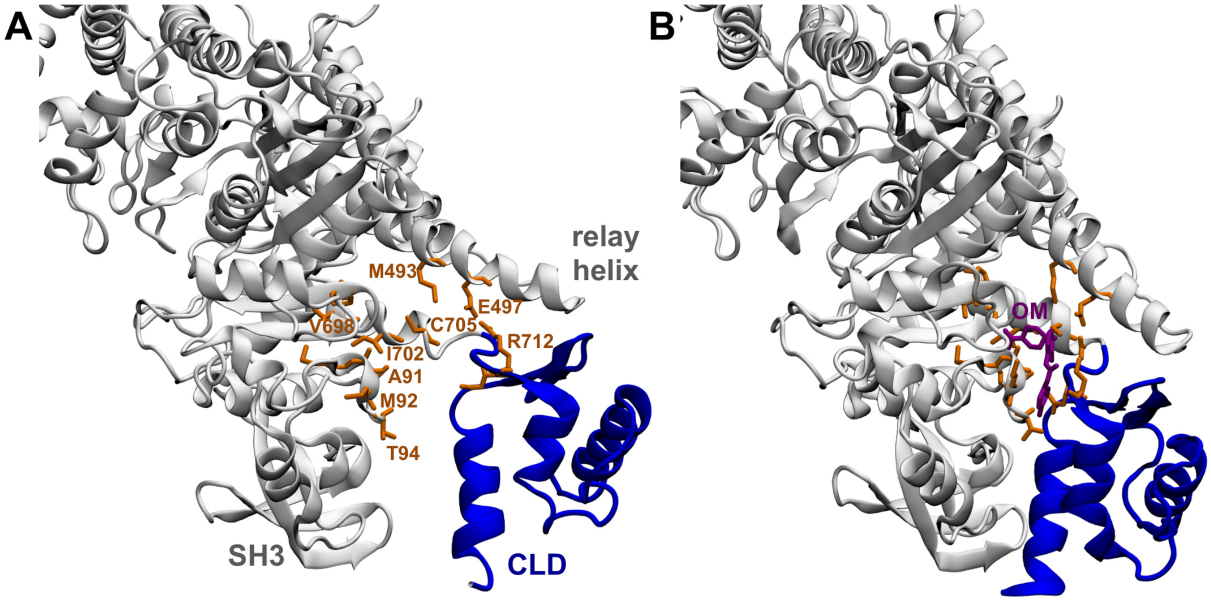

S11 Fig. Comparison of representative structures from Apo and OM-bound trajectories. Cartoon representation of structures sampled at the end of ApoA1 (A) and OMA2 (B) simulations (280 ns). The CLD (blue cartoon) and key residues in the OM-binding region (orange sticks) are highlighted.
