# Supplementary material for: Allosteric modulation of cardiac myosin dynamics by omecamtiv mecarbil
Source: PLoS Comput Biol. 2017 Nov 6;13(11):e1005826. doi: 10.1371/journal.pcbi.1005826 (PMC5690683; doi:10.1371/journal.pcbi.1005826)
Supplement: S12 Fig — DCCM values are reported for Apo (left) and OM-bound (middle) simulations, together with OM-Apo differences (ΔDCCM, right). Differences larger than 0.16 in absolute value are shown in red (positive values) and green (negative values). The ΔDCCM threshold and the colour code are the same as Fig 7. Residues in the modelled loops were not considered in the analysis and are not reported. (PDF) [file pcbi.1005826.s022.pdf]

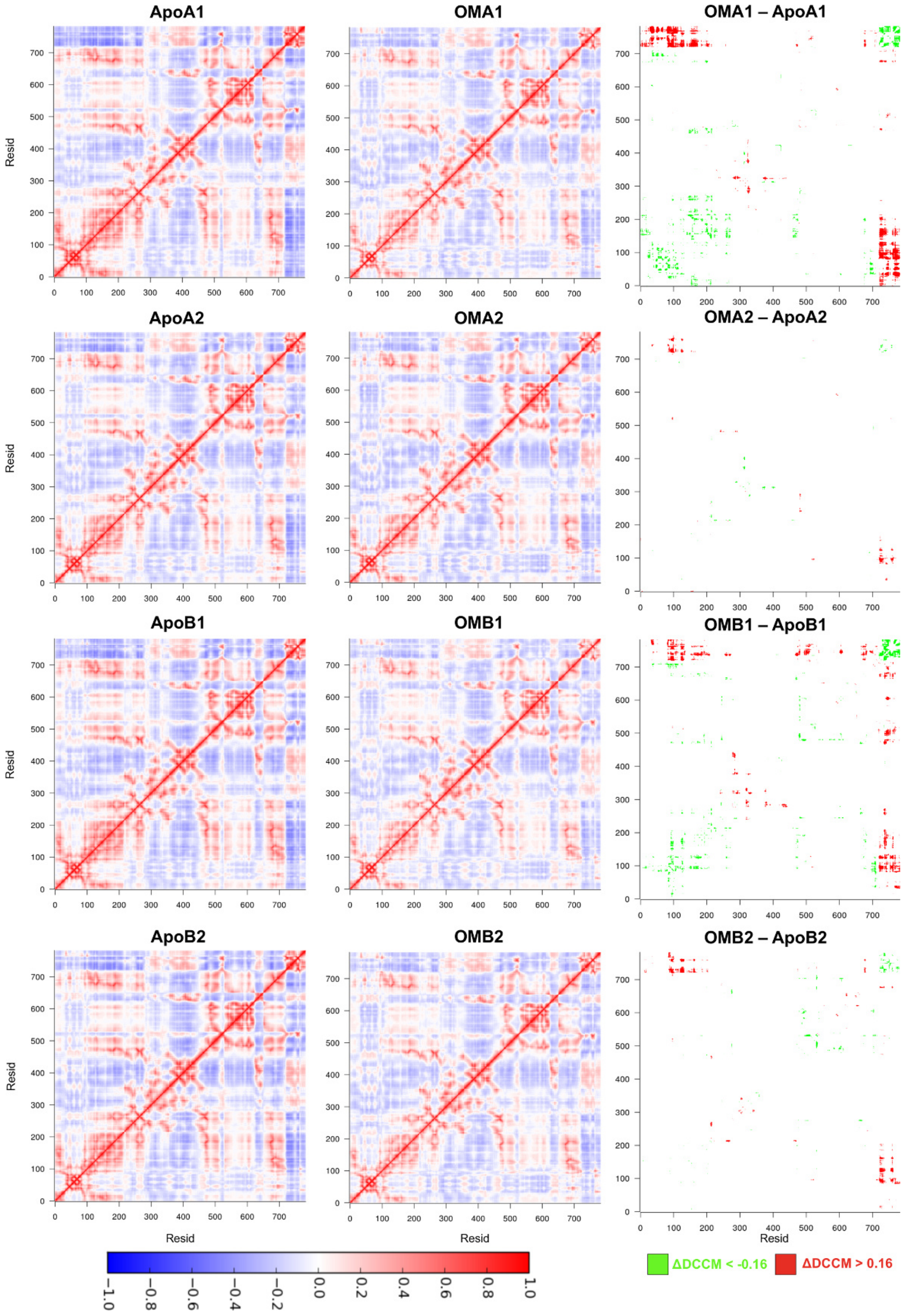

S12 Fig. Dynamical cross-correlation matrices (DCCM). DCCM values are reported for Apo (left) and OM-bound (middle) simulations, together with OM-Apo differences (ΔDCCM, right). Differences larger than 0.16 in absolute value are shown in red (positive values) and green (negative values). The ΔDCCM threshold and the colour code are the same as Fig. 7. Residues in the modelled loops were not considered in the analysis and are not reported.
