# Supplementary material for: Allosteric modulation of cardiac myosin dynamics by omecamtiv mecarbil
Source: PLoS Comput Biol. 2017 Nov 6;13(11):e1005826. doi: 10.1371/journal.pcbi.1005826 (PMC5690683; doi:10.1371/journal.pcbi.1005826)
Supplement: S13 Fig — Plots of the difference between OM and Apo preferential connection scores Δζ calculated using different residues from the OM-binding site as source sites. The source site is indicated on top of each profile. A negative value indicates a stronger preferential connection to the source residue in OM-bound simulations compared to Apo ones. Negative bars are coloured according to the subdomain and grey areas highlight the position of functional regions (see Fig 8 caption for a legend). Residues were selected by clustering the profiles generated for all the residues in the OM-binding site. Cluster representatives are shown here to illustrate the range of variability of the profiles. (PDF) [file pcbi.1005826.s023.pdf]

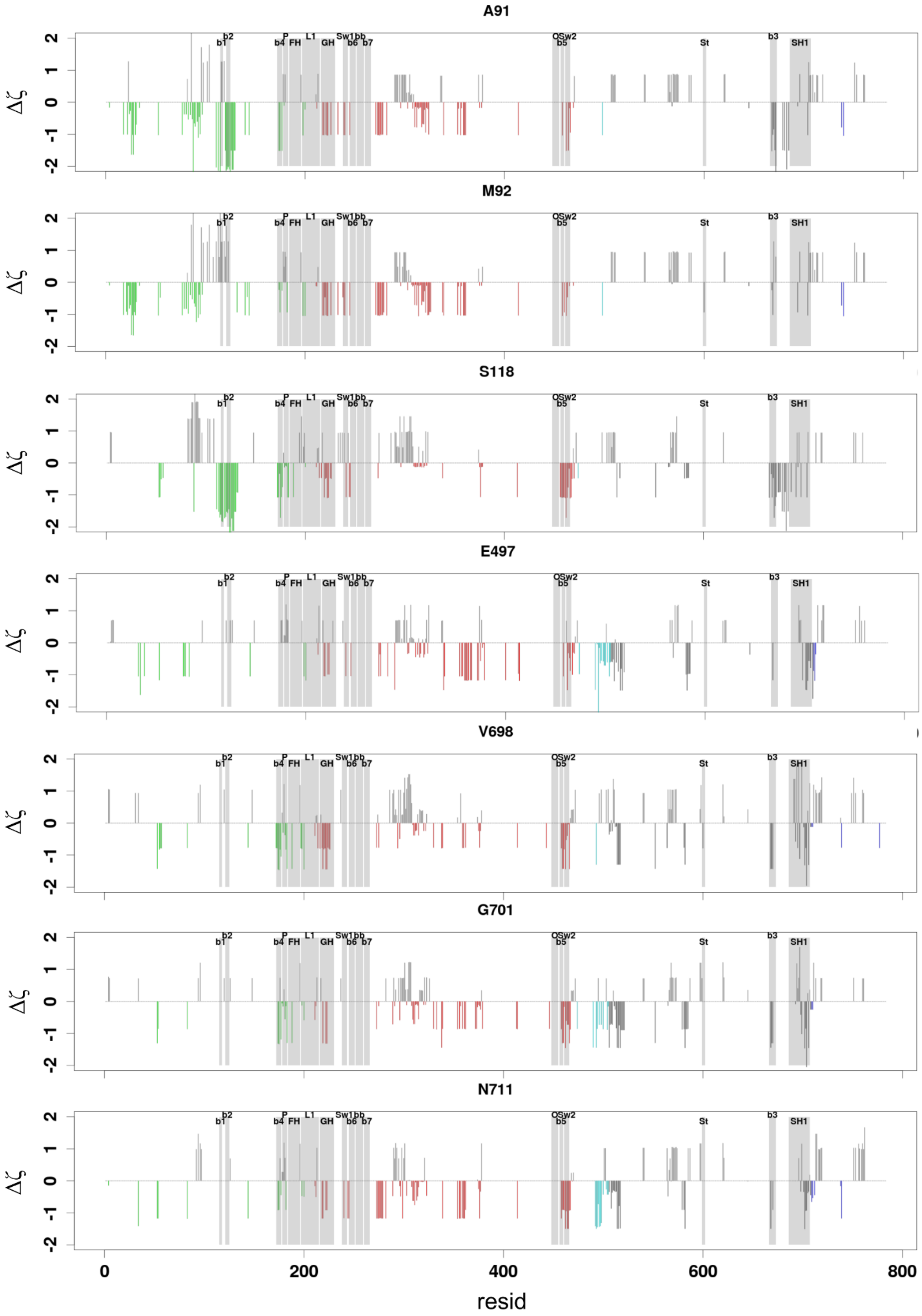

S13 Fig. Preferential connections in the local correlation network between the OM-binding site and functional regions in cMotorD. Plots of the difference between OM and Apo preferential connection scores Δζ calculated using different residues from the OM-binding site as source sites. The source site is indicated on top of each profile. A negative value indicates a stronger preferential connection to the source residue in OM-bound simulations compared to Apo ones. Negative bars are coloured according to the subdomain and grey areas highlight the position of functional regions (see Fig. 8 caption for a legend). Residues were selected by clustering the profiles generated for all the residues in the OM-binding site. Cluster representatives are shown here to illustrate the range of variability of the profiles.
