# Supplementary material for: Allosteric modulation of cardiac myosin dynamics by omecamtiv mecarbil
Source: PLoS Comput Biol. 2017 Nov 6;13(11):e1005826. doi: 10.1371/journal.pcbi.1005826 (PMC5690683; doi:10.1371/journal.pcbi.1005826)
Supplement: S14 Fig — Scores calculated from Apo (top) and OM-bound (bottom) consensus MI matrices are reported using the colour code described in Fig 8 caption. Grey areas highlight the position of functional regions (see Fig 8 caption for a legend). (PDF) [file pcbi.1005826.s024.pdf]

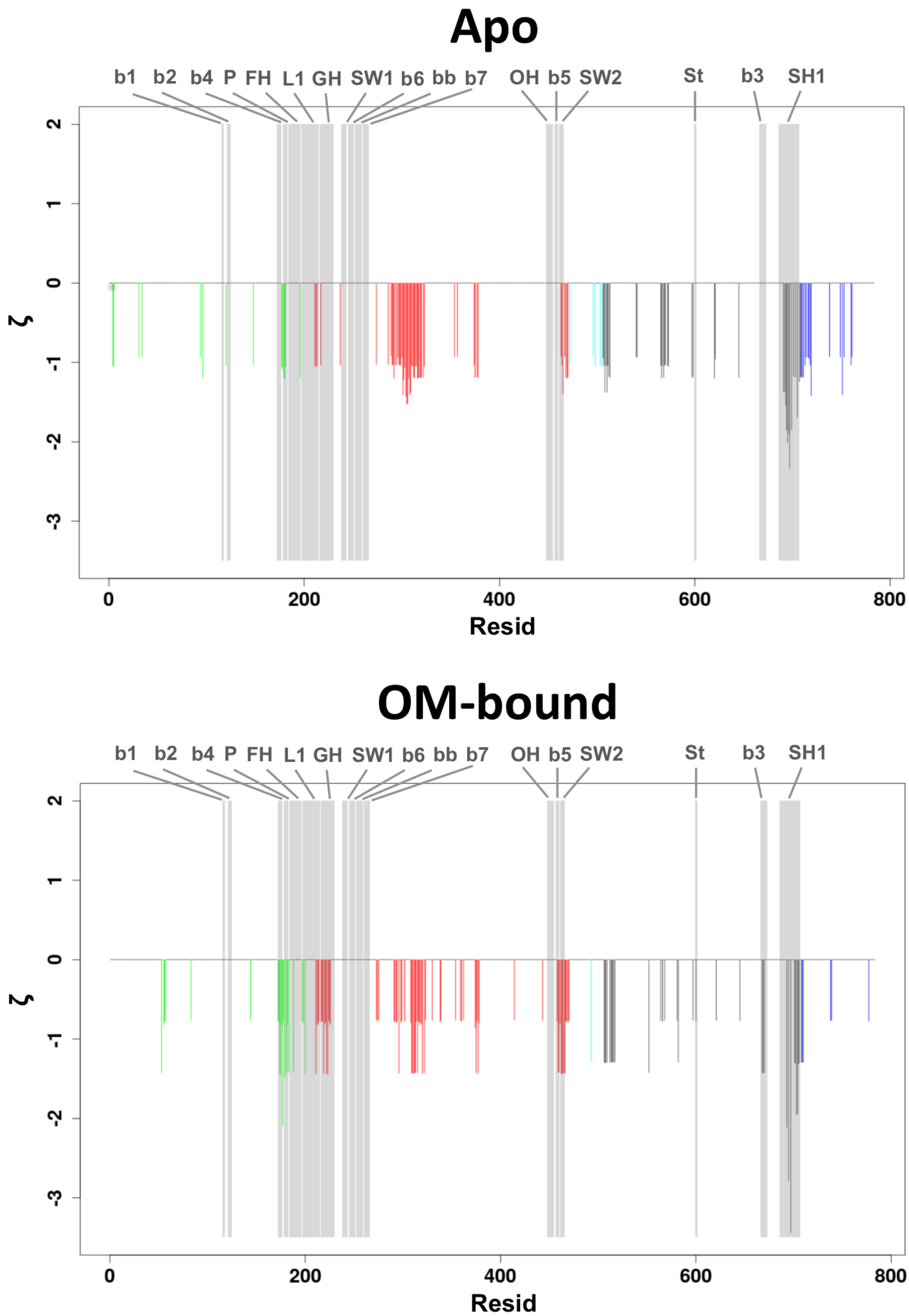

S14 Fig. Profiles of preferential connection scores ζ calculated using V698 as source site. Scores calculated from Apo (top) and OM-bound (bottom) consensus MI matrices are reported using the colour code described in Fig. 8 caption. Grey areas highlight the position of functional regions (see Fig. 8 caption for a legend).
