# Supplementary figures and images for: Allosteric modulation of cardiac myosin dynamics by omecamtiv mecarbil
Source: PLoS Comput Biol. 2017 Nov 6;13(11):e1005826. doi: 10.1371/journal.pcbi.1005826 (PMC5690683; doi:10.1371/journal.pcbi.1005826)

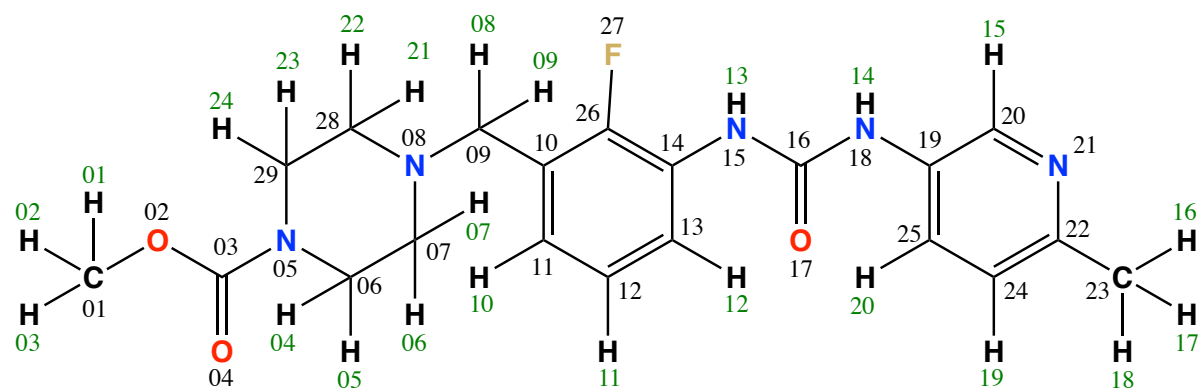

**S15 Fig. Structural formula of omecamtiv mecarbil (OM) with atom numbering.**

Supplement: S15 Fig — (PDF) [file pcbi.1005826.s025.pdf]
