# Supplementary material for: Allosteric modulation of cardiac myosin dynamics by omecamtiv mecarbil
Source: PLoS Comput Biol. 2017 Nov 6;13(11):e1005826. doi: 10.1371/journal.pcbi.1005826 (PMC5690683; doi:10.1371/journal.pcbi.1005826)
Supplement: S16 Fig — The RMSD (Å) values from the initial structure are reported for each Apo (green hues, top panels) and OM-bound (blue hues, bottom panel) simulation. (PDF) [file pcbi.1005826.s026.pdf]

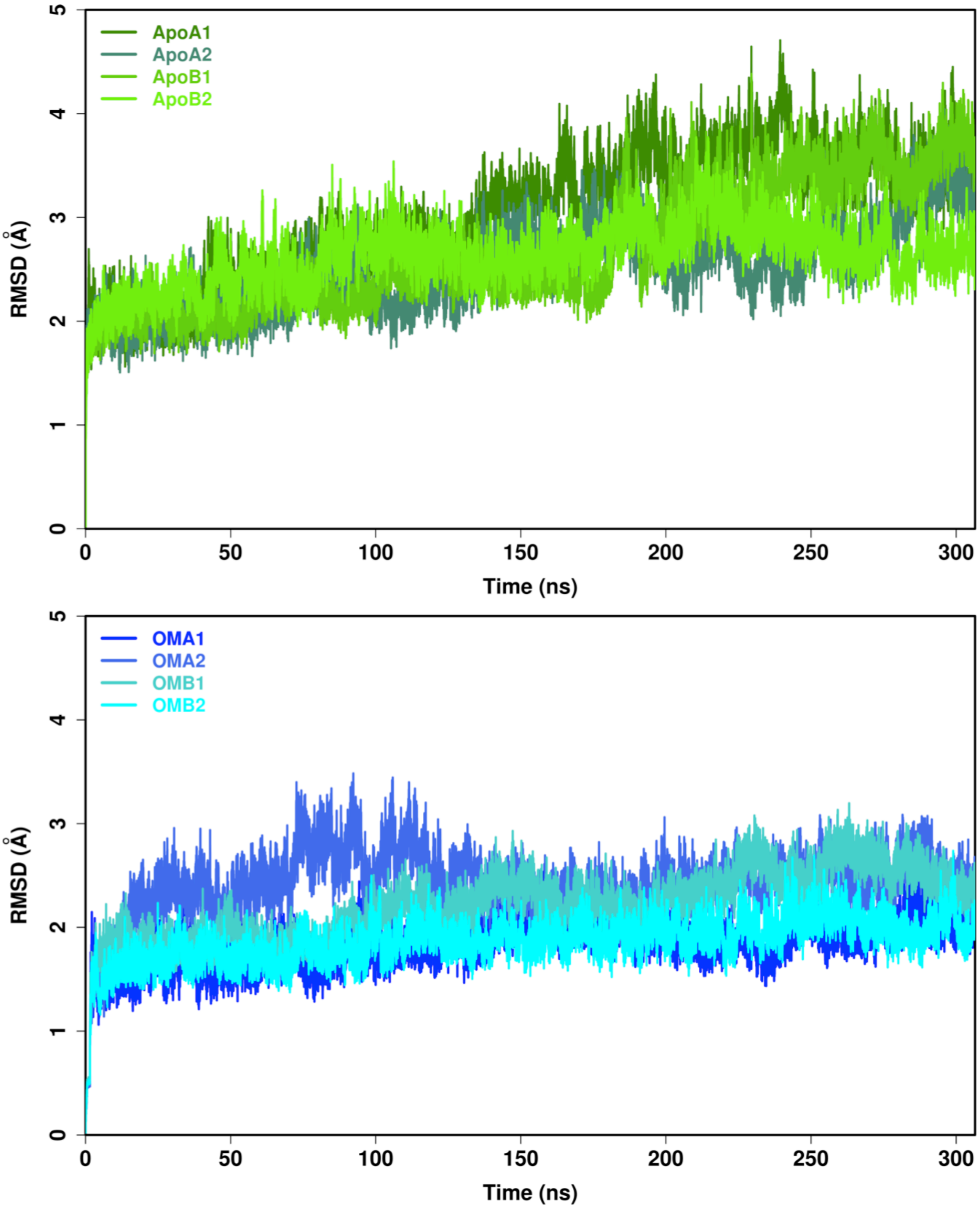

S16 Fig. Time evolution of C$^{\alpha}$ RMSD. The RMSD (Å) values from the initial structure are reported for each Apo (green hues, top panels) and OM-bound (blue hues, bottom panel) simulation.
