# Supplementary material for: Allosteric modulation of cardiac myosin dynamics by omecamtiv mecarbil
Source: PLoS Comput Biol. 2017 Nov 6;13(11):e1005826. doi: 10.1371/journal.pcbi.1005826 (PMC5690683; doi:10.1371/journal.pcbi.1005826)
Supplement: S17 Fig — The DSSP annotation is reported for each simulation, with coloured blocks indicating α-helices (blue), β-strands (red), β-bridges (black), bends (green), turns (yellow), π-helices (purple) and 310-helices (grey). (PDF) [file pcbi.1005826.s027.pdf]

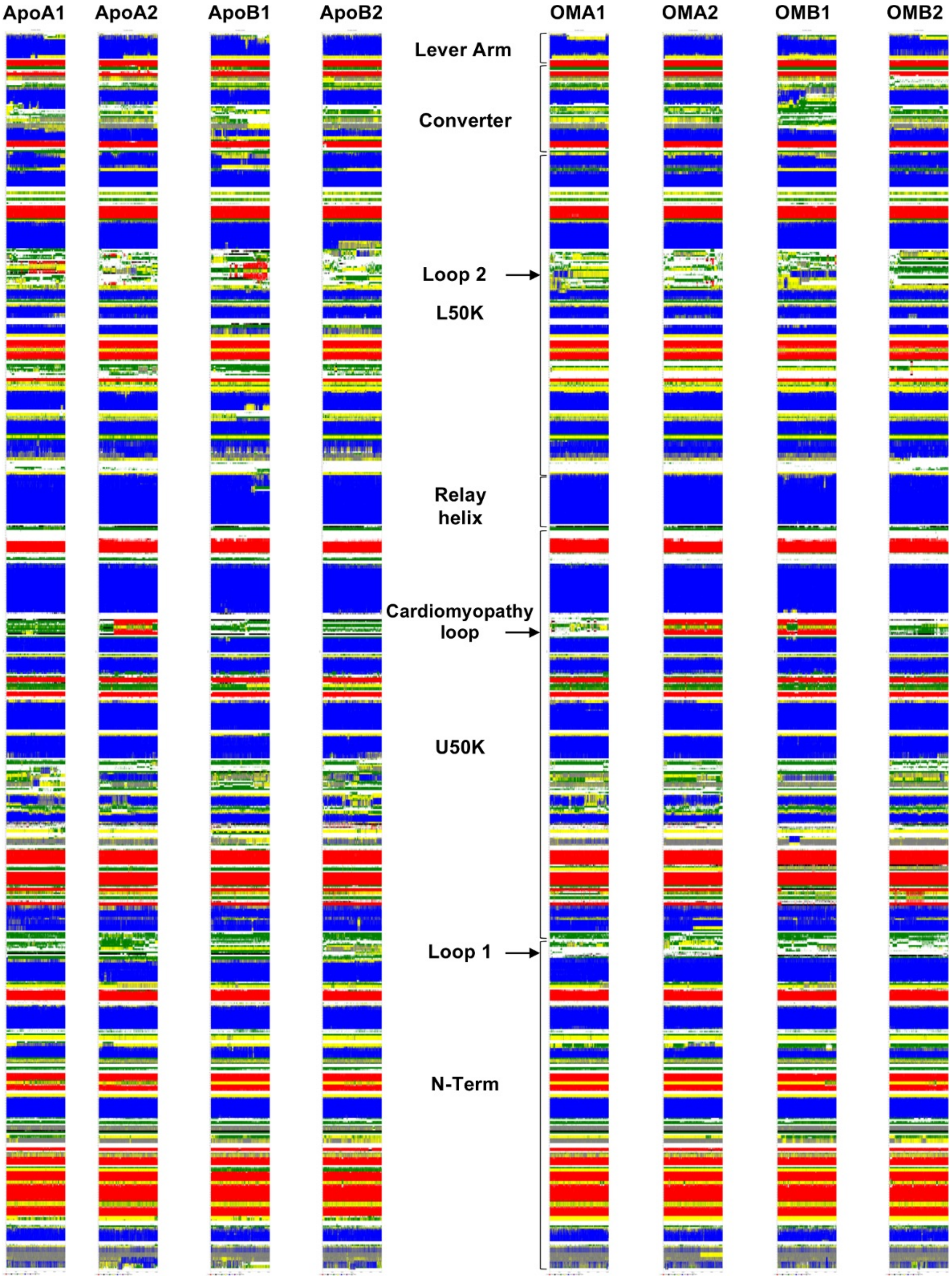

S17 Fig. Time evolution of the secondary structure. The DSSP annotation is reported for each simulation, with coloured blocks indicating α-helices (blue), β-strands (red), β-bridges (black), bends (green), turns (yellow), π-helices (purple) and 3_{10}-helices (grey).
