# Supplementary material for: Allosteric modulation of cardiac myosin dynamics by omecamtiv mecarbil
Source: PLoS Comput Biol. 2017 Nov 6;13(11):e1005826. doi: 10.1371/journal.pcbi.1005826 (PMC5690683; doi:10.1371/journal.pcbi.1005826)
Supplement: S1 Text — (PDF) [file pcbi.1005826.s028.pdf]

Modulation of cardiac myosin dynamics by omecamtiv mecarbil

Shaima Hashem[1], Matteo Tiberti[1], and Arianna Fornili[1,2*]

Supplementary Methods

Loop modelling

The initial structure of the motor domain of the human β-cardiac myosin (resid 1 to 783 of UniProt sequence P12883) was extracted from the Protein Data Bank (PDB) for the OM-bound (PDB ID: 4PA0) and Apo (PDB ID: 4P7H) states [1]. Homology modelling was used to model unsolved parts of the protein in the X-ray structure, which included loops localised either in the actin-binding region or in the converter (S8 Table). Templates were found by running the basic local alignment search tool (BLAST) [2] against the PDB database. Only hits with sequence identity >= 40% were retained (E-value <= 10^{-9}). A multiple sequence alignment of the resulting sequences was performed with M-Coffee [3] using default parameters. Structures from chicken skeletal (PDB ID: 1M8Q) and smooth (PDB ID: 1BR1) muscle were then selected as suitable templates since they contained coordinates for the missing loops with a sequence coverage of 99% and 95% (75% and 45% on average for the loops only) and a sequence identity of 75% and 52% (68% and 33% on average for the loops only), respectively. The chain B structure of the Apo state contained a relatively long gap in the converter structure (resid 717 to 737) that was instead complete in the OM-bound structure chain A, so the latter was used to model ApoB in addition to the templates described above.

MODELLER 9.15 [4] was used to model the missing loops by keeping the rest of the structure unchanged. Since the longer loops were expected to be highly flexible, to make sure that the results obtained from the simulations were not dependent from their specific initial structure, two different loop conformations were generated. These were then used as starting points to run two different replicas for each system, resulting in a total number of eight different MD simulations (S1 Table). The two different models for the loops were generated with two different runs of MODELLER. In the first one (x = 1), all the interatomic restraints were used to model the loops, including both intra- and inter-loop restraints. While intra-loop restraints describe the shape of each loop, inter-loop restraints provide information on their relative orientation. In the second approach (x = 2), the loops were modelled independently by including only intra-loop restraints. For each MODELLER run, 100 models were generated and the one with the lowest DOPE score was selected as the final structure.

MD simulation setup and protocol

All Molecular Dynamics (MD) simulations were performed using GROMACS 4.6.7 [5]. Each system was solvated using a truncated octahedral box of TIP3P water molecules. A minimal distance of 10 Å was set between the protein and the walls of the box. The charge of the ionisable residues was set to that of their standard protonation state at pH 7. The systems were neutralised by adding counter-ions, for a total of ~ 169,000 to 198,000 atoms depending on the specific system, with the octahedron radius (half-distance between two opposite hexagons) varying between 65 and 68.5 Å.

Periodic boundary conditions were applied. The equations of motion were integrated using the leap-frog method with a 2-fs time step. The LINCS [6] algorithm was chosen to constrain all covalent bonds in the protein, while SETTLE [7] was used for water molecules. The Particle Mesh Ewald (PME) [8] method was used for electrostatic interactions, with a 9-Å cutoff for the direct space sums, a 1.2-Å FFT grid spacing, and a 4-order interpolation polynomial for the reciprocal space sums. A 9-Å cutoff was used for van der Waals interactions. Long-range corrections to the dispersion energy were included. The neighbour list for non-covalent interactions was updated every 5 steps.

Each system was minimised through 3 stages with 2000 (positional restraints on heavy atoms) + 3000 steps of steepest descent, followed by 2000 steps of conjugate gradient. Positional restraints on heavy atoms were initially set to 4.8 kcal/mol/Å^2 and they were gradually decreased to 0 in 1.5 ns, while the temperature was increased from 200 to 300 K at constant volume. The system was then allowed to move freely and was subjected to 1-ns equilibration in NVT conditions at T = 300 K. This was followed by a 2-ns equilibration in NPT conditions with T = 300 K and p = 1 bar. For these equilibration steps, the Berendsen [9] algorithm was used for both temperature and pressure regulation with coupling constants of 0.2 and 1 ps, respectively. At last, a 2-ns NPT equilibration was run after switching to the v-rescale thermostat [10] with a coupling constant of 0.1 ps and the Parrinello-Rahman barostat [11] with a coupling constant of 2 ps. Production NPT runs were then performed for 300 ns, saving the coordinates every 1 ps.

The stability of the simulations was checked by monitoring the Root Mean Square Deviation (RMSD) from the initial structure calculated over C$^\alpha$ atoms after best fit superposition and the time evolution of the DSSP secondary structure annotation. The RMSD was below 4 and 3 Å for most part of the Apo and OM-bound simulations, respectively (S16 Fig). The different magnitude of the RMSD in the Apo and OM-bound sets of simulations reflects the different flexibility values observed for the two states. Most of the α-helical and β-strand secondary structure elements were stable throughout the whole simulation in all cases (S17 Fig). A decrease in the helical content was found for the relay and SH1 helix in the ApoB1

simulation, which is consistent with the fact that a) the hinges of the CLD rotation observed in the Apo states are located in these helices (Fig. 3) and b) the ApoB1 simulation presents the most pronounced CLD motions (S1 Fig). The second helix of the converter subdomain showed a decrease in the helical content in the second part of the OMB1 simulation. Some instability in this region might be expected due to the absence of the Regulatory Domain. The cardiomyopathy loop presented a stable β-hairpin structure in three of the simulations (ApoA2, OMA1 and OMB1) and shorter β-bridges in the others. Transient α and β structures were found for Loop2.

OM parametrisation

The GAFF parameters for OM were generated as described in the main text. Before running the production simulations, we performed preliminary runs to test two alternative descriptions for the N05 atom (S15 Fig). Indeed, a sp^2 atom type ('n') was assigned to this nitrogen by antechamber as part of a carbamate group. However, N05 is also part of the piperazine ring and in the X-ray structures it adopts a partially out-of-plane conformation (improper dihedral C03-C06-N05-C29 = 142° and -158° in chain A and chain B, respectively), so we tested whether relaxing its description to a sp^3 atom type provided a smaller deviation from X-ray data. We performed two sets of minimisation+equilibration runs on the OM-bound protein, one with the original antechamber assignment and one with a modified N05 atom type ('n3', the same used for the other nitrogen group in the ring, N08), starting from either the chain A and chain B structure. The sp^2 parameter set produced consistently low RMSD values from the X-ray structure for the piperazine ring at the end of the minimisation ($RMSD_{ring}$ in S10 Table), while the sp^3 description produced a significantly higher value for the chain A structure than the sp^2 one. Moreover, the deviation of dihedral angles involving N05 from the X-ray ones during the equilibration was significantly smaller when using the sp^2 parameters than the sp^3 ones ($RMSD_{dihe}$ in S10 Table), suggesting an excessive amount of flexibility at N05 produced by the sp^3 parameter set. The original antechamber atom type assignment was thus selected for the simulations in this work.

Analysis of MD trajectories

A Principal Component Analysis (PCA) was performed to extract a reduced set of coordinates that can capture the main dynamical features of the simulated systems [12]. The effectiveness of this method is related to the fact that the Principal Components (PCs) with the largest fluctuations have been often shown to describe the most important functional motions of a protein. PCs are obtained as eigenvectors of the covariance matrix of atomic coordinates, calculated as: $c_{ij} = \langle (x_i - \langle x_i \rangle)(x_j - \langle x_j \rangle) \rangle \; (i,j = 1,2,3 \ldots, 3N)$, where x_i indicates an atomic Cartesian coordinate, $\langle x_i \rangle$ its time average and N the number of atoms considered in the analysis. Each eigenvalue measures the amplitude of the fluctuation along

the motion described by the corresponding PC. For each system, the PCA was performed with GROMACS using C^{α} atoms coordinates of snapshots extracted every 100 ps from the production trajectory. The trajectories were then projected onto the PCs associated with the two largest eigenvalues (PC1 and PC2). A PCA was performed also on a pseudo-trajectory obtained by combining the last 100 ns of all the Apo and OM-bound trajectories, with C^{α} atoms coordinates extracted every 100 ps from the single production trajectories and then concatenated.

The identification of dynamic domains was performed with Dyndom [13], which decomposes a protein into regions that move as quasi-rigid domains by comparing two different structures. The domain motions are characterised as domain rotations around hinge axes, with hinge regions located at the interface between the moving domains and the rest of the protein. The DynDom analysis was performed onto the experimental structures representing a post-power stroke (PDB ID: 1SR6) and a pre-power stroke (PDB ID: 1QVI) state to describe the recovery stroke and onto the structures with minimum and maximum PC1 projection value to describe the ApoA1 simulation.

The correlation networks were generated by calculating the dynamical cross-correlation (DCC) [14] matrices as:

$$C_{ij} = \frac{\langle |\mathbf{r}_i - \langle \mathbf{r}_i \rangle| |\mathbf{r}_j - \langle \mathbf{r}_j \rangle| \rangle}{\sqrt{\langle |\mathbf{r}_i - \langle \mathbf{r}_i \rangle|^2 \rangle \langle |\mathbf{r}_j - \langle \mathbf{r}_j \rangle|^2 \rangle}}$$

where $\mathbf{r}_i$ indicates the spatial position of an atom or a centroid of group of atoms from residue *i* and C_{ij} indicates the DCC between residues *i* and *j*. The DCC values can go from -1 to +1, with negative and positive values corresponding to anti-correlated and correlated displacements of the two residues, respectively. For each system, the calculation of DCC values was performed with Wordom [15] using C^{α} atoms coordinates of snapshots extracted from the production trajectory every 100 ps. Multiple matrices were first generated by calculating time averages over 5 ns. Averaging over these matrices produced the final DCC matrix [16].

The calculation and analysis of local correlation networks was performed as described in [17]. Local conformational changes and their correlation were described using the M32K25 [18] Structural Alphabet (SA), i.e. a collection of fragments of 4 consecutive C^{α} atoms representing prototypical backbone conformations. In the SA, each fragment is labelled with a letter. The 3D structure of a protein of N residues can then be represented using a string of length N − 3 by assigning a letter to each 4-residue fragment in the structure. The assignment (encoding) is done by identifying the SA letter that is most similar to the fragment (lowest RMSD). Following this procedure for each structure of an MD trajectory

produces an alignment of structural strings, where each column summarises the conformational states sampled by a protein fragment during the simulation. The correlation of conformational changes in a pair of protein fragments can be calculated as normalized Mutual Information (MI) between the associated columns in the alignment. As previously shown, MI networks can be used to identify allosteric transmission pathways in proteins [17]. In particular, transmission pathways between two regions can be identified by calculating the set of shortest paths connecting them in the MI network.

The SA analysis was performed on C^{α} atoms coordinates extracted every 1 ps. The statistical significance of the MI values was determined by generating a random background distribution of 1000 samples as described in Ref [17]. Only statistically significant values were retained in the final MI matrices. Shortest paths were identified using the Dijkstra algorithm [19] on consensus MI networks derived from all the four simulation of each binding state (Apo and OM-bound). Elements in the consensus MI matrix were calculated as averages over the values obtained from single simulations if they were different from 0 in at least three of the trajectories, while they were set to 0 in all the other cases. Edge weights derived from the consensus MI correlation were taken into account in the calculation of the shortest paths.

Regions preferentially connected to a source residue *a* were determined as described in Ref [17] by calculating the shortest distances between each of the fragments j_a containing *a* and all the protein fragments. The distances were then standardised by calculating their z-scores ζ^{ja} and an overall ζ profile was derived by calculating the minimum over the ζ^{ja} profiles. Fragments with negative ζ values are closer to the source site than the average, so that they can be considered as preferentially connected to it. In our analyses, a ζ profile was calculated from each of the two consensus MI matrices (Apo and OM-bound) and the $\Delta\zeta$ differences were then calculated by subtracting the Apo profile from the OM-bound one.

The matrix of OM-Apo contact changes was derived by first calculating the frequency of inter-residue contacts for each Apo and OM-bound simulation. Two residues were considered to be in contact if the minimum distance calculated over all the pairs of non-hydrogen atoms was < 4 Å. A consensus contact matrix was then calculated from the four simulations of each binding state (Apo and OM-bound). Analogously to the procedure followed for the MI matrix, elements in the consensus contact matrix were calculated as averages over the values obtained from single simulations if they were different from 0 in at least three of the trajectories, while they were set to 0 in all the other cases. The final matrix of OM-Apo contact changes was derived by subtracting the Apo consensus matrix from the OM one and calculating the absolute value, so that elements different from 0 indicate contacts that are either stabilised or destabilised upon OM binding. The values stored in the

matrix go from 0 to 1 and measure the difference in the consensus contact frequency observed in the two sets of simulations. The top 5 shortest paths (i.e. the shortest+suboptimal paths up to a total of 5) between selected endpoints were calculated using a threshold of 0.1 on the frequency difference. Edge weights derived from the contact frequency change were taken into account in the calculation of the shortest paths.

The cluster analysis on OM structures was performed with the 'gromos' clustering method [20] implemented in GROMACS, using a cutoff of 2 Å on structures saved every 100 ps. The four OM-bound trajectories were first concatenated. Each structure in the concatenated trajectory was then fitted to the first one using all the C^{α} atoms in the protein. The RMSD values used for the clustering were then calculated on all OM non-hydrogen atoms.

References

1. Winkelmann D, Forgacs E, Miller MT, Stock AM. Structural basis for drug-induced allosteric changes to human β-cardiac myosin motor activity. Nat Commun. 2015;6:7974.
2. Altschul SF, Gish W, Miller W, Myers EW, Lipman DJ. Basic local alignment search tool. J Mol Biol. 1990;215(3):403-10.
3. Wallace IM, O'Sullivan O, Higgins DG, Notredame C. M-Coffee: combining multiple sequence alignment methods with T-Coffee. Nucleic Acids Res. 2006;34(6):1692-9.
4. Sali A, Blundell TL. Comparative protein modelling by satisfaction of spatial restraints. J Mol Biol. 1993;234(3):779-815.
5. Páll S, Abraham MJ, Kutzner C, Hess B, Lindahl E. Tackling Exascale Software Challenges in Molecular Dynamics Simulations with GROMACS. In: Markidis S., Laure E. (eds) Solving Software Challenges for Exascale. EASC 2014. Lecture Notes in Computer Science, 8759: Springer; 2014. p. 3-27.
6. Hess B, Bekker H, Berendsen HJC. LINCS: a linear constraint solver for molecular simulations. J Comp Chem. 1997;18(12):1463–72.
7. Miyamoto S, A. KP. Settle: An analytical version of the SHAKE and RATTLE algorithm for rigid water models. J Comp Chem. 1992;13(8):952--62.
8. Essmann U, Perera L, Berkowitz ML, Darden T, Lee H, Pedersen LG. A smooth particle mesh Ewald method. J Chem Phys. 1998;103(19):8577--93.
9. Berendsen HJC, Postma JPM, Gunsteren WF, Di Nola A, Haak JR. Molecular dynamics with coupling to an external bath. J Chem Phys. 1984;81(9):3684--90.
10. Bussi G, Donadio D, Parrinello M. Canonical sampling through velocity rescaling. J Chem Phys. 2007;126(1):014101.
11. Parrinello M, Rahman A. Polymorphic transitions in single crystals: A new molecular dynamics method. J Appl Phys. 1981;52(9):7182–90.

12. Daidone I, Amadei A. Essential dynamics: foundation and applications. WIREs Comput Mol Sci. 2012; 2:762-770.

13. Poornam GP, Matsumoto A, Ishida H, Hayward S. A method for the analysis of domain movements in large biomolecular complexes. Proteins. 2009;76(1):201-12.

14. McCammon JA. Protein dynamics. Rep Prog Phys. 1984;47(1):1-46.

15. Seeber M, Felline A, Raimondi F, Muff S, Friedman R, Rao F, et al. Wordom: a user-friendly program for the analysis of molecular structures, trajectories, and free energy surfaces. J Comp Chem. 2011;32(6):1183-94.

16. Tiberti M, Invernizzi G, Papaleo E. (Dis)similarity Index To Compare Correlated Motions in Molecular Simulations. J Chem Theory Comput. 2015;11(9):4404-14.

17. Pandini A, Fornili A, Fraternali F, Kleinjung J. Detection of allosteric signal transmission by information-theoretic analysis of protein dynamics. FASEB J. 2012;26:868-81.

18. Pandini A, Fornili A, Kleinjung J. Structural alphabets derived from attractors in conformational space. BMC Bioinformatics. 2010;11(1):97.

19. Newman M. Networks: An Introduction. Oxford, UK: Oxford University Press; 2010.

20. Daura X, van Gunsteren WF, Mark AE. Peptide Folding: When Simulation Meets Experiment. Angew Chem Int Ed. 1999;38:236-40.
